# Sirtuin1 Suppresses Calcium Oxalate Nephropathy via Inhibition of Renal Proximal Tubular Cell Ferroptosis Through PGC‐1α‐mediated Transcriptional Coactivation

**DOI:** 10.1002/advs.202408945

**Published:** 2024-11-05

**Authors:** Chen Duan, Bo Li, Haoran Liu, Yangjun Zhang, Xiangyang Yao, Kai Liu, Xiaoliang Wu, Xiongmin Mao, Huahui Wu, Zhenzhen Xu, Yahua Zhong, Zhiquan Hu, Yan Gong, Hua Xu

**Affiliations:** ^1^ Department of Radiation and Medical Oncology Zhongnan Hospital of Wuhan University Wuhan Hubei 430071 China; ^2^ Tumor Precision Diagnosis and Treatment Technology and Translational Medicine Hubei Engineering Research Center Zhongnan Hospital of Wuhan University Wuhan Hubei 430071 China; ^3^ Department of Urology Zhongnan Hospital of Wuhan University Wuhan Hubei 430071 China; ^4^ School of Medicine Stanford University Stanford CA 94303 USA; ^5^ Department of Urology Tongji Hospital Tongji Medical College Huazhong University of Science and Technology Wuhan Hubei 430011 China; ^6^ Taikang Center for Life and Medical Sciences Wuhan University Wuhan Hubei 430071 China

**Keywords:** crystal nephropathy, ferroptosis, PGC‐1α, renal proximal tubular cells, Sirt1

## Abstract

Calcium oxalate (CaOx) crystals induce renal tubular epithelial cell injury and subsequent nephropathy. However, the underlying mechanisms remain unclear. In the present study, single‐cell transcriptome sequencing is performed on kidney samples from mice with CaOx nephrocalcinosis. Renal proximal tubular cells are identified as the most severely damaged cell population and are accompanied by elevated ferroptosis. Further studies demonstrated that sirtuin1 (Sirt1) effectively reduced ferroptosis and CaOx crystal‐induced kidney injury in a glutathione peroxidase 4 (GPX4)‐dependent manner. Mechanistically, Sirt1 relies on peroxisome proliferator‐activated receptor gamma coactivator 1α (PGC‐1α) to promote resistance to ferroptosis in the tubular epithelium, and PGC‐1α can recruit nuclear factor erythroid 2‐related factor 2 (NRF2) to the promoter region of GPX4 and co‐activate GPX4 transcription. This work provides new insight into the mechanism of CaOx crystal‐induced kidney injury and identifies Sirt1 and PGC‐1α as potential preventative and therapeutic targets for crystal nephropathies.

## Graphical abstract

Calcium oxalate (CaOx) crystals induce renal tubular epithelial cell injury and subsequent nephropathy. Single‐cell transcriptome sequencing identified the proximal tubular cells (PTCs) as the most significantly affected populations in CaOx‐induced kidney injury. Sirtuin1 (Sirt1) effectively reduces ferroptosis and kidney injury in a glutathione peroxidase 4 (GPX4)‐dependent manner. Sirt1 protects PTCs from CaOx‐induced ferroptosis through the PGC‐1α/NRF2/GPX4 pathway.

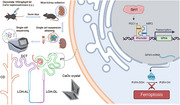

## 1 Introduction

The kidneys are susceptible to crystal formation owing to mineral secretion and urine concentration. Excessive calcium‐containing crystals, mainly calcium oxalate (CaOx), cause tubular injury and intrarenal inflammation, resulting in crystal deposition, nephrocalcinosis, and urolithiasis.^[^ ^1^, ^2^, ^3^ ^]^ The cell‐crystal interactions induce tubular epithelial cell (TEC) death and epithelial barrier breakdown. However, the underlying mechanism remains unclear. Recently, a growing number of non‐apoptotic cell deaths, including necroptosis, ferroptosis, and cytoproptosis, have been identified.^[^ ^4^, ^5^, ^6^ ^]^ Anders et al. originally reported that the cytotoxicity of crystals was associated with receptor‐interacting serine‐threonine kinase 3/mixed lineage kinase domain‐like pathway‐related necroptosis. However, blocking necroptosis had a limited effect on reducing the cytotoxicity of these crystals.^[^ ^7^ ^]^ Subsequently, calcium oxalate was proposed to induce acute kidney injury through a peptidylprolyl isomerase F‐dependent mitochondrial permeability transition.^[^ ^8^ ^]^ More studies have suggested that crystal exposure induces TEC ferroptosis,^[^ ^9^ ^]^ but the underlying molecular mechanisms remain to be elucidated.

Ferroptosis, characterized by excessive iron‐related lipid peroxidation, drives tissue damage caused by cell disintegration and necroinflammation. Susceptibility to ferroptosis depends on intrinsic antioxidant pathways, among which the cysteine/glutathione (GSH)/glutathione peroxidase (GPX) 4 axis plays a major role in limiting ferroptosis.^[^ ^10^ ^]^ GPX4 transfers intracellular lipid peroxides to their corresponding alcohols by consuming GSH. GPX4 deficiency causes excessive lipid peroxidation, while its overexpression results in a strong resistance to ferroptosis.^[^ ^11^ ^]^ Therefore, targeting the imbalance in intracellular lipid peroxidation may be a promising strategy to reduce crystal‐induced ferroptosis and tissue damage.

NAD^+^‐dependent deacetylase sirtuin1 (Sirt1) plays an important role in modulating energy homeostasis, cell metabolism, and oxidative stress via the deacetylation of histones and a variety of non‐histone proteins.^[^ ^12^, ^13^ ^]^ As a robust regulator of cellular metabolism, Sirt1 can reverse lipid metabolic disorders.^[^ ^14^, ^15^ ^]^ Importantly, Sirt1 was also reported to inhibit lipid peroxidation and proposed as a novel target for the regulation of ferroptosis.^[^ ^16^, ^17^, ^18^ ^]^ Our previous studies demonstrated that metformin reduced CaOx‐induced kidney injury via activating Sirt1.^[^ ^19^ ^]^ In this study, we explored the effects of Sirt1 on crystal‐induced TEC ferroptosis and its underlying mechanisms.

Peroxisome proliferator‐activated receptor gamma coactivator (PGC)‐1α, one of the most characterized downstream targets of Sirt1, is activated by Sirt1‐mediated deacetylation.^[^ ^20^, ^21^, ^22^ ^]^ PGC‐1α is mainly expressed in the heart, kidney, brain, muscle, and adipose tissue, functioning as a regulator of lipid metabolism and oxidative stress through increased transcription of various antioxidant enzymes including GPXs.^[^ ^23^ ^]^ As a transcriptional co‐activator, PGC‐1α interacts with a variety of transcription factors, such as NRF1/2, ERRα, and PPARs.^[^ ^24^ ^]^ Previous studies have demonstrated that PGC‐1α has a renoprotective effect and that the deficiency of PGC‐1α leads to the aggravation of cisplatin‐induced tubular injury.^[^ ^25^ ^]^ Consequently, we hypothesized that PGC‐1α promotes the transcription of the important intracellular lipid peroxide scavenger GPX4 to alleviate lipid peroxidation in TECs and prevent CaOx‐induced ferroptosis and kidney injury.

In this study, we performed single‐cell transcriptome sequencing and identified the proximal tubular cells (PTCs) as the most damaged cell population following CaOx‐induced kidney injury. The transcriptional levels of GPX4 were lower in injured PTCs. We further demonstrated that Sirt1 protects PTCs from CaOx‐induced ferroptosis through the PGC‐1α/NRF2/GPX4 pathway. Moreover, conditional knockout of Sirt1 in tubular epithelium accelerated CaOx‐induced kidney injury and crystal deposition in vivo, while PGC‐1α and NRF2 agonists could reverse these effects. Our work deepens the understanding of the mechanism underlying CaOx crystal‐induced kidney injury and identifies Sirt1 and PGC‐1α as promising targets for treating crystal nephropathies.

## 2 Results

### 2.1 Ferroptosis in PTCs is Responsible for CaOx‐Induced Kidney Injury

**Figure 1:**
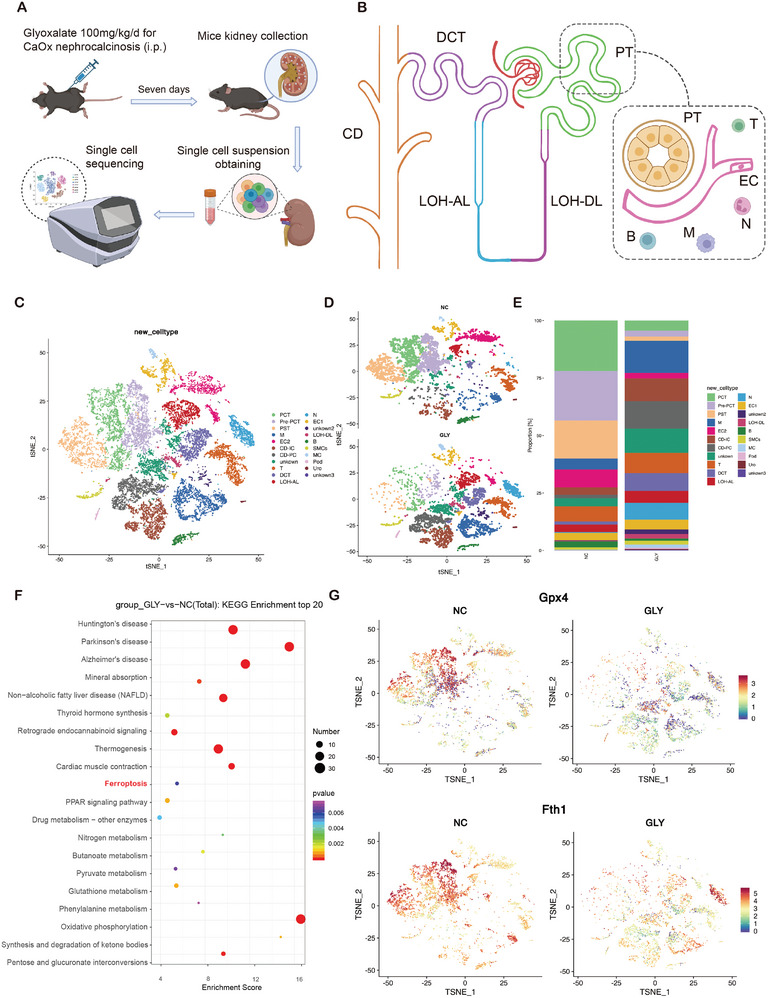
Single cell sequence revealed the change of ferroptosis associated signaling in proximal convoluted tubule of CaOx nephrocalcinosis mouse model. A) Strategy for inducing CaOx nephrocalcinosis in mice. B) Schematic illustration of the mice kidney. PCT, proximal convoluted tubule; PST, proximal straight tubule; LOH‐DL, descending limb of loop of Henle; LOH‐AL, ascending limb of loop of Henle; DCT, distal convoluted tubule; CD‐PC, collecting duct‐principal cell; CD‐IC, collecting duct‐intercalated cell; Uro, urothelium; MC, mesangial cell; Pod, podocyte; SMC, smooth muscle cell; EC, endothelial cell; M, macrophage; T, T cell; N, Neutrophil; B, B cell. C) t‐SNE representation of 21 cell types in mice kidney. D) t‐SNE representation of the distribution across subclusters in the NC and GLY groups. E) Percentage of cells per cluster. F) KEGG pathway analysis of DEGs in PCTs between the NC and GLY groups. G) Expression of GPX4 and Fth1 on t‐SNE representation.

To better understand the mechanism of CaOx‐induced crystal deposition and kidney injury, we performed single‐cell transcriptome sequencing using kidney samples from a mouse model of CaOx‐induced nephrocalcinosis induced by intraperitoneal injection of glyoxylate (GLY, **Figure** **1**A,B). Unsupervised clustering identified 21 cell populations (Figure 1C). Based on the reported markers across cell types in the mouse kidney, we annotated 18 clusters; the remaining three clusters were not annotated (Figure S1A and Table S1, Supporting Information). Cluster 1 was identified as the proximal convoluted tubule (PCT) cells with five well‐known markers (Slc5a2, Hnf4a, Slc5a12, Snhg11, and Slc7a7; Figure S1B,C, Supporting Information).^[^ ^26^ ^]^ When counting the proportion of each cell type in the GLY and control groups, PTCs, including PCT, pre‐PCT, and proximal straight tubule (PST) cells, were identified as the most significantly affected populations (Figure 1D,E). The proximal tubule, which connects to the parietal layer of Bowman's capsule, is an important part of glomerular filtrate reabsorption. Most of the nutrients, water, and ions in the ultrafiltrate were reabsorbed in this segment, especially the PCT closest to Bowman's capsule. Therefore, PCT cells were influenced more by CaOx crystals and were the focus of our subsequent analyses. To investigate the mechanisms underlying the significantly decreased PCT levels in the GLY group, we identified differentially expressed genes (DEGs) in PCT cells and performed KEGG gene set enrichment analysis (Figure S1D, Supporting Information). Ferroptosis was suggested to be activated in PCT cells of GLY‐treated mice (Figure 1F), which might account for PCT cell damage. Among the DEGs associated with ferroptosis, the important regulators GPX4 and Fth1 were markedly decreased (Figure 1G). These results suggest that CaOx leads to PTC loss by inducing ferroptosis and that inhibiting ferroptosis is a promising strategy to treat CaOx‐induced kidney injury.

### 2.2 Ferroptosis Was the Main Form of CaOx‐Induced Tubular Epithelium Death

**Figure 2:**
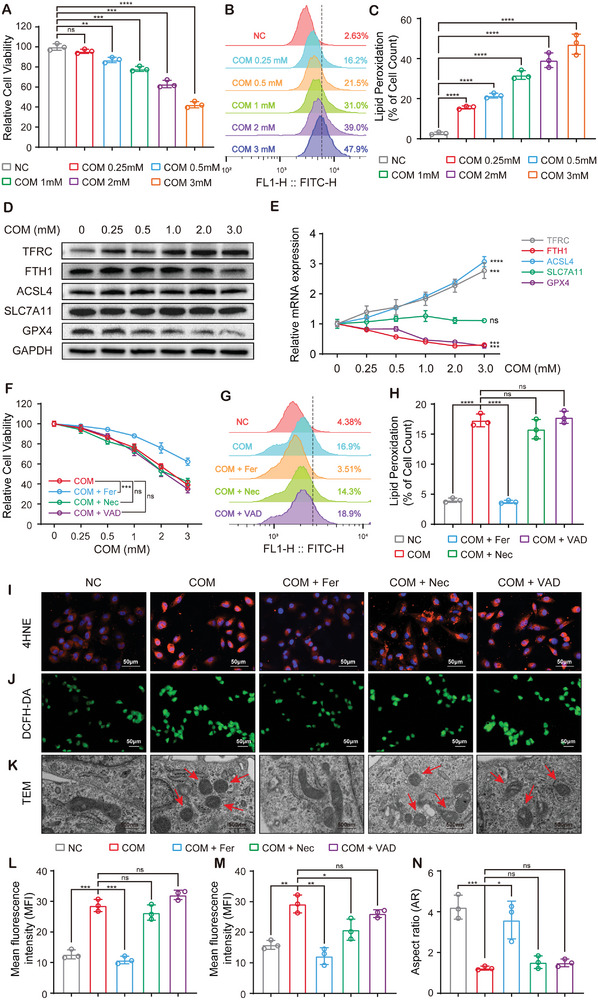
Ferroptosis is the main form of CaOx‐induced cell death.A) Cell viability of COM‐treated HK‐2 cells was evaluated by CCK‐8. B,C) Lipid peroxidation in COM‐treated HK‐2 cells was determined by BODIPY C11 staining and flow cytometry. Immunoblotting D) and qPCR E) revealed the expression of ferroptosis associated markers. F) Cell viability was measured in HK‐2 cells treated with COM and Fer/Nec/VAD. G,H) Lipid peroxidation in HK‐2 cells treated with COM and Fer/Nec/VAD was determined by BODIPY C11 staining and flow cytometry. I,L) Level of 4HNE was detected by immunofluorescence. J,M) Intracellular ROS generation was detected by DCFH‐DA staining. K) The mitochondrial microstructure was observed under TEM. The red arrows indicate the morphologically abnormal mitochondria, characterized by mitochondrial shrinkage and disorganized cristae. N) Quantification of mitochondrial shape transitions by aspect ratio. Data were presented as mean ± SD, *n* = 3, and P value was determined by one‐way (C, E, H, L‐N) or two‐way (A, F) ANOVA. ^*^ *p* < 0.05; ^**^ *p* < 0.01; ^***^ *p* < 0.001; ^****^ *p* < 0.0001.

To confirm the findings of the single‐cell transcriptome sequencing of in vivo samples, we treated human proximal tubule HK‐2 cells with CaOx monohydrate (COM) in vitro. CCK8 assay showed that the viability of HK‐2 cells decreased gradually with increasing COM concentrations (**Figure** **2**A). Consistently, BODIPY C11 staining and flow cytometry showed that COM treatment increased lipid peroxidation, which is the main characteristic of ferroptosis (Figure 2B,C). The detection of lipid peroxidation end‐products (MDA and 4HNE) and lipid ROS levels in HK‐2 cells further demonstrated the relationship between ferroptosis and COM cytotoxicity (Figure 2D; Figure S2A–E, Supporting Information). However, COM treatment did not affect the level of Fe^2+^ in HK‐2 cells (Figure S2F,G, Supporting Information). Immunoblotting and qPCR confirmed that, although COM treatment did not affect the expression of SLC7A11, it altered the expression levels of multiple ferroptosis‐associated genes, including GPX4, FTH1, TFRC, and ACSL4. (Figure 2D,E; Figure S2H, Supporting Information).

When pretreated HK‐2 cells with ferroptosis inhibitor Ferrostatin‐1 (Fer, 1µM), necroptosis inhibitor Necrostatin‐1 (Nec, 10µM) or apoptosis inhibitor Z‐VAD‐FMK (VAD, 10µM) followed by COM treatment, Fer reversed COM‐induced cell deaths, while Nec and VAD had limited effects (Figure 2F). BODIPY C11 staining and flow cytometry also showed that Fer, but not Nec or VAD, significantly inhibited the COM‐induced accumulation of lipid ROS (Figure 2G,H). MDA detection and 4HNE immunofluorescence showed the same results, accompanied by the reversed expression of ferroptosis‐associated genes (Figure 2I,L; Figure S2I–L, Supporting Information). In addition, ferroptosis inhibitors had better rescue effects on COM‐induced intracellular ROS levels than necroptosis and apoptosis inhibitors (Figure 2J,M). Transmission electron microscopy (TEM) illustrated that Fer, but not Nec or VAD, reversed COM‐induced mitochondrial shrinkage and increased membrane density in HK‐2 cells (Figure 2K,N). These results suggested that intracellular lipid peroxidation‐related ROS and end‐product levels increased under the stimulation of CaOx crystals, resulting in mitochondrial damage and cell death. The inhibition of ferroptosis effectively reduced the cytotoxicity of CaOx crystals, indicating that ferroptosis was the main cause of CaOx‐induced tubular epithelium death.

### 2.3 Inhibition of Ferroptosis Reduced GLY‐Induced CaOx Crystal Deposition and Kidney Injury

**Figure 3:**
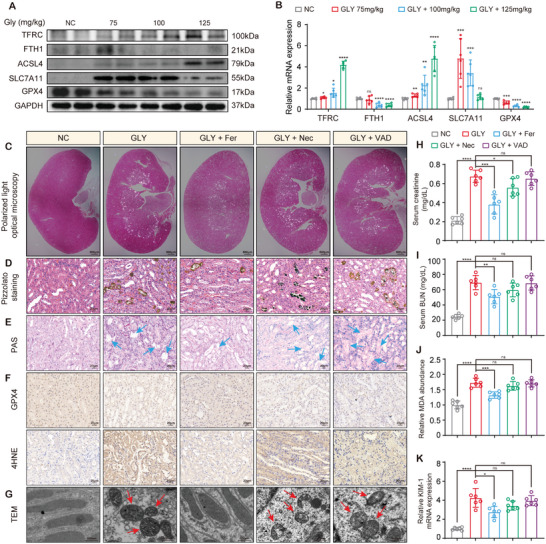
Inhibition of ferroptosis reduced GLY‐induced tubular injury and CaOx crystal deposition. Immunoblotting A) and qPCR B) revealed the expression of ferroptosis associated markers in kidney of mice with CaOx nephrocalcinosis. Mice were pretreated with Fer/Nec/VAD and then induced to establish a model of CaOx nephrocalcinosis. The deposition of CaOx was observed under a polarized light microscopy C) and evaluated by Pizzolato staining D). E) PAS staining was used for the scoring of tubular injury. The blue arrows highlight damaged renal tubules, characterized by tubular atrophy or dilation, cast formation, and brush edge loss. F) IHC staining was performed to test the level of GPX4 and 4HNE in mouse kidney. G) The changes of mitochondrial morphology in tubular epithelium were observed by TEM. The red arrows indicate the morphologically abnormal mitochondria, characterized by mitochondrial shrinkage and disorganized cristae. H, I) The levels of serum creatinine and BUN were detected to assess kidney function. J) MDA in tissues of mouse kidney was quantified. K) The expression of KIM‐1 mRNA in tissues of mouse kidney was determined by qPCR. Data were presented as mean ± SD, *n* = 6, and P value was determined by one‐way ANOVA (B, H‐K). ^*^ *p* < 0.05; ^**^ *p* < 0.01; ^***^ *p* < 0.001; ^****^ *p* < 0.0001.

We investigated the effect of ferroptosis inhibition on CaOx‐induced kidney injury in vivo. C57/B6J mice were intraperitoneally injected with GLY (75, 100, or 125 mg kg^−1^) to establish a CaOx nephrocalcinosis model. As expected, the degree of CaOx crystal deposition and renal tubule lesions was aggravated by an increase in the GLY concentration (Figure S3A–D, Supporting Information). Immunohistochemistry showed that GLY treatment reduced the expression of GPX4 and FTH1 while increasing the level of 4HNE in a dose‐dependent manner (Figure S3E,F, Supporting Information). Similar to the in vitro results, GPX4 and FTH1 were significantly down‐regulated in the GLY group (**Figure** **3**A,B; Figure S3G, Supporting Information). However, pretreatment with Fer reduced GLY‐induced crystal deposition in the kidney tissues, as shown by hematoxylin and eosin (HE) staining (observed by polarized light microscopy) and Pizzolato staining (Figure 3C,D, Figure S3H,I, Supporting Information). PAS staining confirmed that the inhibition of ferroptosis attenuated tubular atrophy or dilation, cast formation, and brush edge loss caused by CaOx crystals (Figure 3E; Figure S3J, Supporting Information). Furthermore, Fer reversed the CaOx‐induced accumulation of lipid peroxidation end products (4HNE) and partially restored GPX4 expression (Figure 3F; Figure S3K, Supporting Information). Transmission electron microscopy (TEM) showed that CaOx‐induced mitochondrial shrinkage was prevented by Fer treatment (Figure 3G; Figure S3L, Supporting Information). Ferroptosis inhibitors also effectively decreased serum creatinine and blood urea nitrogen (BUN) and restored renal function (Figure 3H,I). In addition, Fer treatment reduced malondialdehyde (MDA) content and expression of kidney injury markers in renal tissues (Figure 3J,K). These results suggest that inhibition of ferroptosis might be a promising strategy for reducing CaOx‐induced crystal deposition and kidney injury.

### 2.4 Sirt1 Inhibited CaOx‐Induced Ferroptosis in Tubular Epithelium

**Figure 4:**
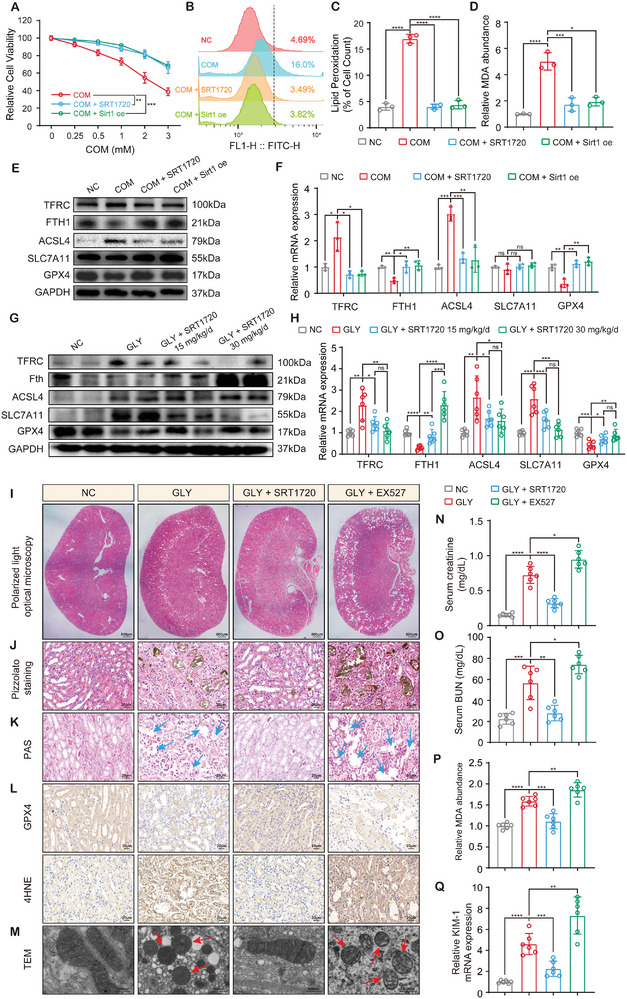
Sirt1 inhibited CaOx‐induced ferroptosis in tubular epithelium. A) Sirt1 activation and overexpression restored the cell viability of COM‐treated HK‐2 cells evaluated by CCK‐8. B, C) Lipid peroxidation in HK‐2 cells was determined by BODIPY C11 staining and flow cytometry. D) Sirt1 activation and overexpression reduced the level of MDA in COM‐treated HK‐2 cells. Immunoblotting E) and qPCR F) revealed the expression of ferroptosis associated markers in HK‐2 cells. In vivo, mice were pretreated with SRT1720/EX527 and then induced to establish a model of CaOx nephrocalcinosis. Immunoblotting G) and qPCR H) revealed the expression of ferroptosis associated markers in HK‐2 cells. The deposition of CaOx was observed under a polarized light microscopy I) and evaluated by Pizzolato staining J). K) PAS staining was used for the scoring of tubular injury. The blue arrows highlight damaged renal tubules. L) IHC staining was performed to test the level of GPX4 and 4HNE in mouse kidney. M) The changes of mitochondrial morphology in tubular epithelium were observed by TEM. The red arrows indicate the morphologically abnormal mitochondria, characterized by mitochondrial shrinkage and disorganized cristae. N, O) The serum levels of creatinine and BUN were detected to assess kidney function. P) MDA in tissues of mice kidney was quantified. Q) The expression of KIM‐1 mRNA in tissues of mice kidney was determined by qPCR. Data were presented as mean ± SD, *n* = 3 for in vitro experiments and *n* = 6 for in vivo experiments. P value was determined by one‐way (C, D, F, H, N‐Q) or two‐way (A) ANOVA. ^*^ *p* < 0.05; ^**^ *p* < 0.01; ^***^ *p* < 0.001; ^****^ *p* < 0.0001.

Since Sirt1 has been reported to relieve intracellular oxidative stress and improve cellular lipid metabolism disorders,^[^ ^13^, ^14^, ^15^ ^]^ and our previous studies demonstrated the renoprotective role of Sirt1 in CaOx nephrocalcinosis,^[^ ^19^ ^]^ we hypothesized that Sirt1 is a novel target for inhibiting CaOx‐induced ferroptosis and kidney injury. As expected, both pharmacological and genetic activation of Sirt1 significantly restored cell viability and reduced COM‐induced lipid peroxidation in HK‐2 cells (**Figure** **4**A–D). Consistently, Sirt1 agonists SRT1720 and Sirt1 overexpression both decreased 4HNE staining and intracellular ROS levels, accompanied by the restoration of mitochondrial morphology (Figure S4A–F, Supporting Information). Therefore, Sirt1 rescues CaOx‐induced lipid peroxidation and ferroptosis in HK‐2 cells. Interestingly, Sirt1 reversed COM‐reduced GPX4/FTH1 expression and COM‐induced TFRC/ACSL4 expression but did not affect SLC7A11 expression (Figure 4E,F; Figure S4G, Supporting Information). These results suggest that Sirt1 has broad benefits for ferroptosis resistance in HK‐2 cells and that Sirt1 might protect the tubular epithelium against CaOx‐induced ferroptosis in multiple ways.

To further verify the role of Sirt1 in CaOx‐induced ferroptosis, renal injury, and crystal deposition in vivo, C57/B6J mice were pretreated with Sirt1 agonists (SRT1720, 15 or 30 mg kg^−1^/d) and inhibitors (EX527, 10 mg k^−1^g/day) by intraperitoneal injection. Consistently, Sirt1 reversed the GLY‐reduced GPX4/FTH1 expression and GLY‐induced TFRC expression in the kidney tissues (Figure 4G,H; Figure S4H, Supporting Information). SRT1720 significantly attenuated renal CaOx crystal deposition, whereas EX527 aggravated the progression of crystal deposition (Figure 4I,J; Figure S4I,J, Supporting Information). PAS staining further revealed that SRT1720 alleviated CaOx‐induced tubular injury, while EX527 exacerbated it (Figure 4K; Figure S4K, Supporting Information). In addition, immunohistochemistry confirmed that SRT1720 significantly attenuated CaOx‐induced accumulation of the lipid peroxidation marker (4HNE) and restored GPX4 expression in the tubular epithelium, whereas EX527 showed the opposite effects (Figure 4L; Figure S4L, Supporting Information). Transmission electron microscopy (TEM) showed that SRT1720 restored normal mitochondrial morphology in renal tubular epithelial cells (Figure 4M; Figure S4M, Supporting Information). Moreover, Sirt1 reversed the GLY‐induced increases in serum creatinine and BUN levels (Figure 4N,O). Furthermore, SRT1720 reduced, but EX527 aggravated, MDA levels and expression of the renal injury marker KIM‐1 in the renal tissues of CaOx nephrocalcinosis mice (Figure 4P,Q). Thus, in vivo, data confirmed that Sirt1 exerts significant renoprotective effects by inhibiting ferroptosis, kidney injury, and CaOx crystal deposition.

### 2.5 Sirt1 Inhibited CaOx‐Induced Ferroptosis Through GPX4 in Tubular Epithelium

**Figure 5:**
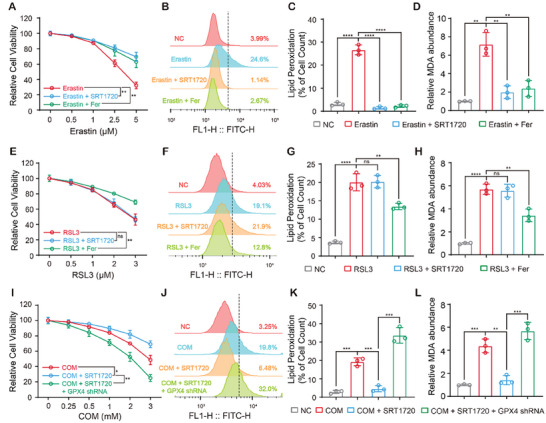
Sirt1 inhibited CaOx‐induced ferroptosis in tubular epithelium dependent on GPX4.HK‐2 cells were treated with increasing concentrations of erastin, along with 5 µM SRT1720 or 1 µM Fer for 24h. A) Cell viability of HK‐2 cells was evaluated by CCK‐8. B, C) Lipid peroxidation in HK‐2 cells was determined by BODIPY C11 staining and flow cytometry. D) Level of MDA in HK‐2 cells treated with erastin, along with SRT1720 or Fer for 24 h. (E) HK‐2 cells were treated with increasing concentrations of RSL3, along with 5 µM SRT1720 or 1 µM Fer for 24 h. Cell viability of HK‐2 cells was evaluated by CCK‐8. F, G) Lipid peroxidation in HK‐2 cells was determined by BODIPY C11 staining and flow cytometry. H) Level of MDA in HK‐2 cells treated with erastin, along with SRT1720 or Fer for 24 h. I) Cell viability of Sh^ctrl^ and GPX4^sh^ HK‐2 cells treated with increasing concentrations of COM and SRT1720 was evaluated by CCK‐8. J, K) Lipid peroxidation in Sh^ctrl^ and GPX4^sh^ HK‐2 cells was determined by BODIPY C11 staining and flow cytometry. L) Level of MDA in Sh^ctrl^ and GPX4^sh^ HK‐2 cells treated with increasing concentrations of COM and SRT1720 for 24 h. Data were presented as mean ± SD, *n* = 3, and P value was determined by one‐way (C, D, G, H, K, L) or two‐way (A, E, I) ANOVA. ^*^ *p* < 0.05; ^**^ *p* < 0.01; ^***^ *p* < 0.001; ^****^ *p* < 0.0001.

To understand the role and mechanism of Sirt1 in suppressing ferroptosis, we examined its effects on ferroptosis induced by classical ferroptosis inducers in HK‐2 cells. Interestingly, both SRT1720 and Fer treatments significantly reduced erastin‐induced cell death and lipid peroxidation (**Figure** **5**A–D), whereas SRT1720 failed to abolish RSL3‐induced cell death and lipid peroxidation (Figure 5E–H). As a class I ferroptosis inducer, erastin induced ferroptosis mainly via targeting the Xc^−^ system to limit cystine transport, while class II ferroptosis inducer (such as RSL3) directly blocked the enzyme activity of GPX4 to induce ferroptosis.^[^ ^27^ ^]^ These results strongly suggest that Sirt1 regulates ferroptosis in a GPX4‐dependent manner, which is consistent with our previous finding that Sirt1 dramatically elevates GPX4 expression. We further confirmed that GPX4 deficiency abrogated the inhibitory effects of Sirt1 on ferroptosis and lipid peroxidation (Figure 5I–L), demonstrating that Sirt1 enhances ferroptosis resistance in HK‐2 cells by regulating GPX4.

### 2.6 Sirt1 Inhibited Ferroptosis in Tubular Epithelium via a PGC‐1α/GPX4 Pathway

**Figure 6:**
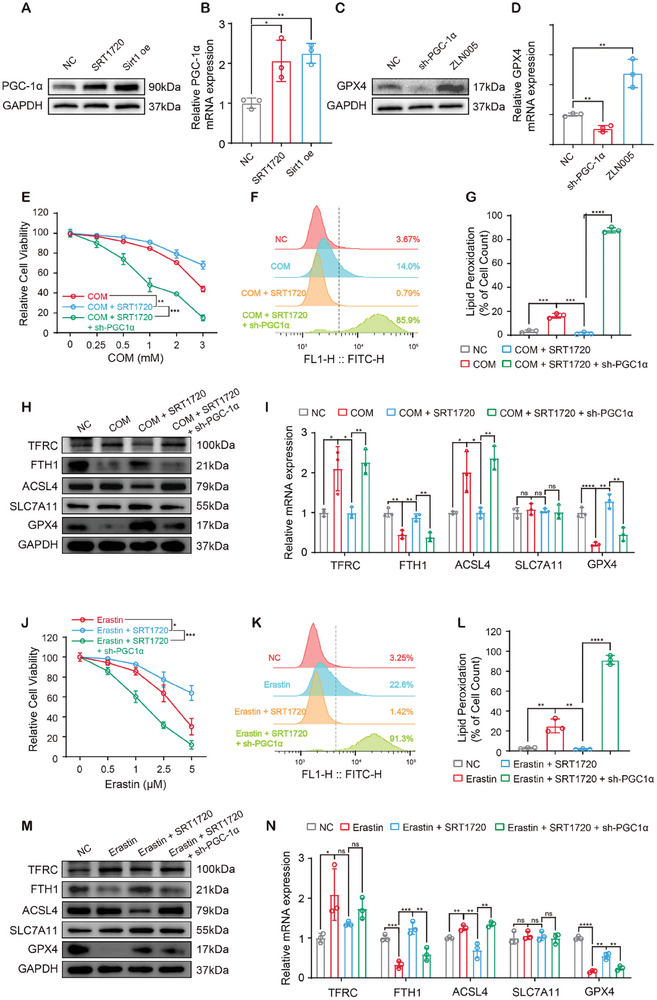
Sirt1 inhibited ferroptosis in tubular epithelium through the PGC‐1α/GPX4 pathway.A, B) Sirt1 activation and overexpression increased the protein and mRNA expression of PGC‐1α. C, D) The protein and mRNA levels of GPX4 in Sh^ctrl^ and PGC‐1α^sh^ HK‐2 cells treated by ZLN005. E) Cell viability of Sh^ctrl^ and PGC‐1α^sh^ HK‐2 cells treated with increasing concentrations of COM and SRT1720 was evaluated by CCK‐8. F, G) Lipid peroxidation in Sh^ctrl^ and PGC‐1α^sh^ HK‐2 cells was determined by BODIPY C11 staining and flow cytometry. Immunoblotting H) and qPCR I) revealed the expression of ferroptosis associated markers in Sh^ctrl^ and PGC‐1α^sh^ HK‐2 cells treated by COM and SRT1720. J) Cell viability of Sh^ctrl^ and PGC‐1α^sh^ HK‐2 cells treated with increasing concentrations of erastin and SRT1720 was evaluated by CCK‐8. K, L) Lipid peroxidation in Sh^ctrl^ and PGC‐1α^sh^ HK‐2 cells was determined by BODIPY C11 staining and flow cytometry. Immunoblotting M) and qPCR N) revealed the expression of ferroptosis associated markers in Sh^ctrl^ and PGC‐1α^sh^ HK‐2 cells treated by erastin and SRT1720. Data were presented as mean ± SD, *n* = 3, and P value was determined by one‐way (B, D, G, I, L, N) or two‐way (E, J) ANOVA. ^*^ *p* < 0.05; ^**^ *p* < 0.01; ^***^ *p* < 0.001; ^****^ *p* < 0.0001.

PGC‐1α is a transcriptional coactivator and regulates the expression of a variety of antioxidant enzymes, including GPXs.^[^ ^23^ ^]^ As a major downstream target of Sirt1, PGC‐1α is involved in the regulatory functions of Sirt1 on lipid metabolism. Thus, we hypothesized that Sirt1 upregulated GPX4 expression via activating PGC‐1α, thereby inhibiting CaOx‐induced ferroptosis in renal tubular epithelial cells. Both pharmacological and genetic activation of Sirt1 promoted PGC‐1α expression (**Figure** **6**A,B; Figure S5A–D, Supporting Information), accompanied by PGC‐1α‐promoted GPX4 expression (Figure 6C,D; Figure S5E, Supporting Information). Surprisingly, PGC‐1α deficiency not only blocked the protective effects of SRT1720 against cell death and lipid peroxidation caused by CaOx crystals but also led to unstoppable massive lipid peroxidation and cell death of HK‐2 cells on the basis of the cytotoxicity of COM (Figure 6E–G; Figure S5F, Supporting Information). Immunoblotting and qPCR showed that Sirt1 upregulated GPX4 and FTH1expression and downregulated TFRC and ACSL4 expression in a PGC‐1α‐dependent manner (Figure 6H,I; Figure S5G, Supporting Information). These results indicated that PGC‐1α deficiency dramatically elevated the sensitivity of HK‐2 cells to ferroptosis and that Sirt1 inhibited CaOx‐induced ferroptosis of HK‐2 cells via the activation of PGC‐1α.

We further investigated whether Sirt1 inhibited erastin‐induced ferroptosis through PGC‐1α as well. Erastin obviously changed the morphology of HK‐2 cells (smaller and rounder), and Sirt1 restored the normal morphology of HK‐2 cells, which was reversed by PGC‐1α deficiency (Figure S5H, Supporting Information). Consistently, PGC‐1α deficiency blocked the protective effects of SRT1720 against ferroptosis caused by erastin and caused a more extensive lipid peroxidation and cell death (Figure 6J–L; Figure S5I, Supporting Information). Moreover, PGC‐1α mediated the regulatory effects of Sirt1 on the expression of ferroptosis markers in erastin‐treated HK‐2 cells (Figure 6M,N; Figure S5J, Supporting Information). Together, Sirt1‐promoted resistance to ferroptosis was dependent on PGC‐1α, which was a key regulator of ferroptosis in HK‐2 cells.

### 2.7 PGC‐1α and NRF2 Coactivated GPX4 Transcription

**Figure 7:**
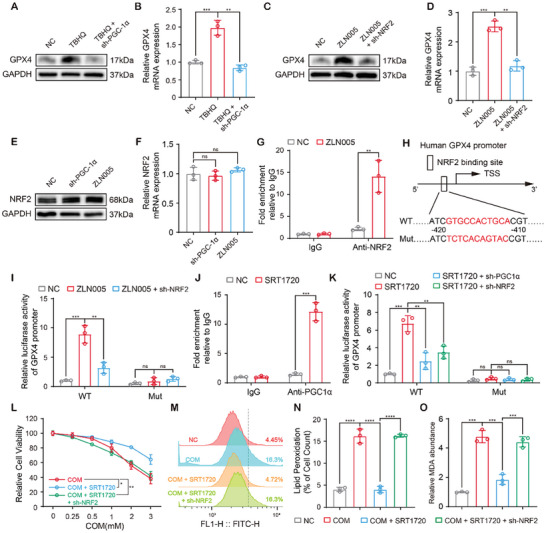
PGC‐1α and NRF2 coactivated GPX4 transcription.A, B) The protein and mRNA levels of GPX4 in Sh^ctrl^ and PGC‐1α^sh^ HK‐2 cells treated by TBHQ. C, D) The protein and mRNA levels of GPX4 in Sh^ctrl^ and NRF2^sh^ HK‐2 cells treated by ZLN005. E, F) The protein and mRNA levels of NRF2 in Sh^ctrl^ and PGC‐1α^sh^ HK‐2 cells treated by ZLN005. G) ChIP analysis of the direct binding of NRF2 to the promoter region of GPX4. H) The NRF2 binding site was muted in the promoter region of GPX4. I) WT and NRF2 binding site mutant GPX4 promoters were used to establish the luciferase reporter plasmids. J) ChIP analysis of the PGC‐1α binding to the promoter region of GPX4. K) Sh^ctrl^, NRF2^sh^ and PGC‐1α^sh^ HK‐2 cells were transfected with the WT and mutant luciferase reporter plasmids and treated with ZLN005. L) Cell viability of Sh^ctrl^ and NRF2^sh^ HK‐2 cells treated with increasing concentrations of COM and SRT1720 was evaluated by CCK‐8. (M, N) Lipid peroxidation in Sh^ctrl^ and NRF2^sh^ HK‐2 cells was determined by BODIPY C11 staining and flow cytometry. O Level of MDA in Sh^ctrl^ and NRF2^sh^ HK‐2. Data were presented as mean ± SD, *n* = 3, and P value was determined by Student's t test (G, J), one‐way (B, D, F, N, O) or two‐way (I, K, L) ANOVA. ^*^ *p* < 0.05; ^**^ *p* < 0.01; ^***^ *p* < 0.001; ^****^ *p* < 0.0001.

Given that the inhibitory effects of Sirt1 on ferroptosis were GPX4‐dependent and that the inductive effects of Sirt1 on GPX4 expression were PGC‐1α‐dependent, we next explored the mechanisms by which PGC‐1α regulated GPX4. Among the transcription factors interacting with PGC‐1α, the activation of PGC‐1α/NRF2 signaling was reported to protect nerve cells by increasing the production of antioxidant enzymes.^[^ ^28^ ^]^ NRF2 is a well‐characterized ferroptosis suppressor that promotes GPX4 transcription^[^ ^29^, ^30^ ^]^ but the signal that activates NRF2 remains unclear. Here, we hypothesized that PGC‐1α activated GPX4 transcription via recruiting NRF2 to the promoter region. Indeed, PGC‐1α deficiency abolished the expression of GPX4 induced by NRF2 agonist TBHQ (**Figure** **7**A,B; Figure S6A, Supporting Information). On the other hand, the promotion of ZLN005 (a PGC‐1α agonist) on GPX4 expression was also dependent on NRF2 (Figure 7C,D; Figure S6B–E, Supporting Information). In addition, PGC‐1α had no effect on NRF2 expression (Figure 7E,F; Figure S6F, Supporting Information). These data suggested that PGC‐1α and NRF2 collaborated to activate GPX4 transcription.

NRF2 generally regulates gene transcription by binding to antioxidant response elements (AREs, 5′‐TGACnnnGC‐3′) in the promoter regions (Figure S6G, Supporting Information). Using the JASPAR database, we identified a potential NRF2 binding site 410 to 420 bp upstream of the transcription start site (TSS) of GPX4 (Figure S6H, Supporting Information). ChIP assays confirmed that NRF2 is bound to the promoter region of GPX4 and that ZLN005 increased the enrichment of NRF2 in the promoter region (Figure 7G). Luciferase assays demonstrated that ZLN005 significantly increased GPX4 promoter‐luciferase activity, whereas NRF2 deficiency reversed this effect, and ARE mutation completely abolished the GPX4 promoter‐luciferase activity (Figure 7H,I).

As a transcriptional coactivator, PGC‐1α does not bind to the promoter region in a sequence‐specific manner. Using a series of ChIP‐qPCR primers for the promoter region of GPX4, we confirmed the binding of PGC‐1α 400–600 bp upstream of the TSS of the GPX4 gene, which also overlapped with the ARE mentioned above. In addition, SRT1270 increased PGC‐1α enrichment in the promoter region of GPX4 (Figure 7J). Luciferase assays demonstrated that Sirt1‐induced GPX4 transcription was dependent on both PGC‐1α and NRF2 (Figure 7K). NRF2 deficiency blocked the protective effects of SRT1720 against ferroptosis and lipid peroxidation in HK‐2 cells (Figure 7L–O). These results provided further evidence that PGC‐1α recruits NRF2 to co‐activate GPX4 expression and inhibits CaOx‐induced ferroptosis in tubular epithelium.

### 2.8 Sirt1 Inhibited CaOx‐Induced Ferroptosis, Crystal Deposition and Kidney Injury Through PGC‐1α/NRF2 Signaling

**Figure 8:**
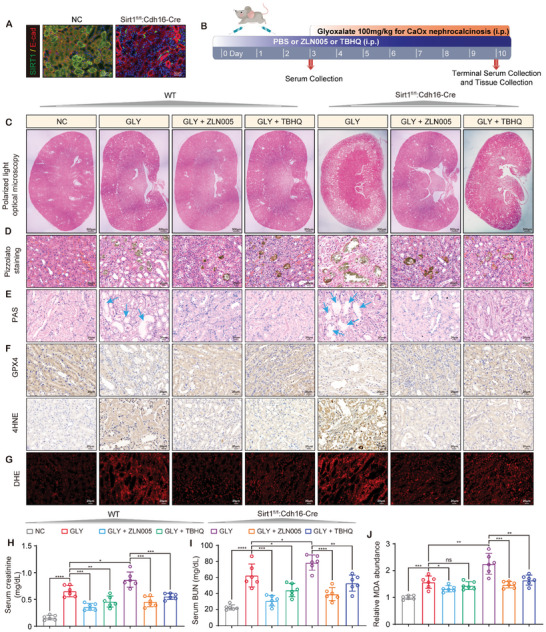
Sirt1 inhibited CaOx‐induced ferroptosis, crystal deposition and kidney injury through PGC‐1α/NRF2 signaling.A) The conditional knockout of Sirt1 in tubular epithelium in mouse kidney. B) Schematic of establishment of CaOx nephrocalcinosis model and treatment of ZLN005 and TBHQ. The deposition of CaOx was observed under a polarized light microscopy C) and evaluated by Pizzolato staining D). E) PAS staining was used for the scoring of tubular injury. The blue arrows highlight damaged renal tubules. F) IHC staining was performed to test the level of GPX4 and 4HNE in mouse kidney. G) The levels of renal ROS were detected by DHE staining. H, I) The serum levels of creatinine and BUN were detected to assess kidney function. J) MDA in tissues of mice kidney was quantified. Data were presented as mean ± SD, *n* = 6, and P value was determined by one‐way ANOVA (H‐J). ^*^ *p* < 0.05; ^**^ *p* < 0.01; ^***^ *p* < 0.001; ^****^ *p* < 0.0001.

To explore the role of Sirt1 expression in the tubular epithelium during CaOx‐induced ferroptosis in vivo, we generated Sirt1^fl/fl^/Cdh16‐cre mice and confirmed the conditional knockout of Sirt1 (Sirt1cKO) in the tubular epithelium (**Figure** **8**A; Figure S7A–D, Supporting Information). The wild‐type and Sirt1cKO mice were pretreated with ZLN005 (15 mg kg^−1^/day) and TBHQ (10 mg kg^−1^/day) for 3 days, followed by an intraperitoneal injection of GLY (Figure 8B). We found that Sirt1cKO exacerbated GLY‐induced crystal deposition in the kidney tissues, whereas both ZLN005 and TBHQ showed inhibitory effects on crystal deposition (Figure 8C,D; Figure S7E,F, Supporting Information). PAS staining further revealed that Sirt1cKO worsened GLY‐induced tubular injury, while both ZLN005 and TBHQ partially mitigated tubular injury induced by GLY and Sirt1cKO (Figure 8E; Figure S7G, Supporting Information). Immunohistochemistry confirmed that Sirt1cKO significantly increased the accumulation of lipid peroxidation end products (4HNE), but reduced GPX4 expression, whereas ZLN005 and TBHQ reversed these effects (Figure 8F; Figure S7H, Supporting Information). DHE staining showed that Sirt1cKO aggravated GLY‐induced renal ROS generation, whereas activation of PGC‐1α and NRF2 attenuated the generation of renal ROS induced by GLY and Sirt1cKO (Figure 8G; Figure S7I, Supporting Information). In addition, we measured serum creatinine and BUN levels and found that Sirt1cKO caused a more significant decline in renal function after GLY treatment, whereas ZLN005 and TBHQ partially restored renal function (Figure 8H,I). Finally, Sirt1cKO exacerbated GLY‐induced MDA accumulation and elevated KIM‐1 expression in kidney tissues, which were effectively reduced by ZLN005 and TBHQ treatment (Figure 8J; Figure S7J, Supporting Information).

In conclusion, Sirt1 enhances the resistance of renal tubular epithelium to ferroptosis induced by CaOx crystals through the PGC‐1α/NRF2/GPX4 pathway, thereby reducing CaOx‐induced kidney damage and further crystal deposition. Sirt1 and PGC‐1α agonists can be used as a promising treatment for CaOx nephrocalcinosis.

## 3 Discussion

CaOx crystals are significantly cytotoxic to the renal tubular epithelium, causing cell death and crystal deposition in the tubulointerstitium, ultimately leading to nephrocalcinosis and even renal failure.^[^ ^1^, ^31^ ^]^ Previous studies reported that CaOx crystals induced massive ROS production and caused oxidative stress damage to the renal tubular epithelium. Additionally, several antioxidants have been reported to reduce oxidative damage to the tubular epithelium and crystal deposition.^[^ ^19^, ^32^ ^]^ These data implied that protecting renal tubular epithelium from CaOx‐induced damage might be the key to preventing further crystal deposition and kidney injury. According to their morphological structure, distribution, and function, renal tubules can be divided into proximal tubules, loops of Henle, distal tubules, and collecting ducts. Through single‐cell transcription sequencing, we identified PTCs as the main damaged cell group in mouse kidneys. Furthermore, we found that ferroptosis signaling pathways were activated in the PTCs of GLY‐treated mice, which might have resulted in CaOx‐induced cell death and tubular injury. Our results verified that ferroptosis was the main form of CaOx‐induced cell death, and that ferroptosis inhibition reduced CaOx‐induced kidney injury and crystal deposition. Notably, we recognized that other cell death pathways, such as pyroptosis and autophagy, may also contribute to CaOx‐induced renal injury. Pyroptosis, through inflammatory mechanisms, could exacerbate kidney injury in CaOx nephropathy.^[^ ^33^, ^34^ ^]^ Similarly, impairment of autophagy has been shown to increase oxidative stress and cell damage, potentially contributing to the progression of CaOx nephropathy.^[^ ^35^, ^36^ ^]^ However, these mechanisms require further investigation.

GPX4 is one of the most important targets in the regulation of ferroptosis, and its deficiency leads to excessive accumulation of intracellular lipid peroxides.^[^ ^37^, ^38^ ^]^ Our studies revealed that GPX4 expression was decreased in the renal tubular epithelium of CaOx nephrocalcinosis mice, accompanied by accumulated lipid peroxides. CaOx decreases GPX4 expression and increases lipid peroxidation in HK‐2 cells. Interestingly, CaOx also affects the expression of various ferroptosis regulators. Specifically, FTH1 expression decreased upon CaOx treatment, whereas the expression levels of ASCL4 and TFRC increased. CaOx crystals may induce the production of lipid peroxides, impair the ability of cells to remove lipid peroxides and increase the generation of iron‐related ROS, thereby promoting ferroptosis. However, the mechanism by which CaOx crystals regulate these ferroptosis regulators requires further investigation.

Our study demonstrates the potent inhibitory effects of Sirt1 on lipid peroxidation and ferroptosis in the tubular epithelium. Surprisingly, the protective effects of Sirt1 against ferroptosis differed depending on the inducer used. Sirt1 markedly inhibits erastin but not RSL3‐induced ferroptosis. RSL3 directly targeted and inhibited the enzymatic activity of GPX4 to trigger ferroptosis, while erastin induced ferroptosis via targeting the Xc^−^ system to inhibit GSH synthesis and inhibit VDAC2/3 to reduce NADH oxidation.^[^ ^27^ ^]^ Therefore, erastin induced ferroptosis without directly targeting GPX4, while RSL3 caused complete inactivation of GPX4. Our data imply that the inhibitory effects of Sirt1 on ferroptosis are GPX4‐dependent. In addition, Sirt1 activation significantly upregulates FTH1 expression and downregulates ACSL4 expression, both of which have been reported to regulate ferroptosis.^[^ ^39^, ^40^, ^41^, ^42^ ^]^ To explore the effects of Sirt1 on CaOx‐induced ferroptosis in the tubular epithelium in vivo, we generated Sirt1^fl/fl^; Cdh16‐Cre mice. Conditional Sirt1 knockout in PTCs further reduced GPX4 expression and increased CaOx‐induced renal tubular injury and crystal deposition. These results highlight the critical role of mitigating renal tubular epithelial damage and cell death in preventing CaOx‐induced kidney injury and crystal deposition.

As the most characteristic downstream target of Sirt1, PGC‐1α was demonstrated to play an important role in regulating ferroptosis in this study. PGC‐1α deficiency completely reversed cytoprotection of Sirt1 and triggered irreversible lipid peroxidation and ferroptosis in HK‐2 cells. These results indicated that PGC‐1α was a key regulator of ferroptosis resistance in HK‐2 cells, and the inhibitory effects of Sirt1 on ferroptosis were PGC‐1α‐dependent. Notably, GPX4 deficiency did not cause such dramatic accumulation of lipid peroxidation and cell death in HK‐2 cells compared with PGC‐1α deficiency. PGC‐1α might be an extremely important controller of cellular resistance to ferroptosis in the renal tubular epithelium, regulating the transcription of various genes involved in ferroptosis. We further demonstrated that Sirt1 regulated the expression of GPX4, FTH1, TFRC, and ACSL4 in a PGC‐1α‐dependent manner. As a transcriptional coactivator, PGC‐1α regulates gene transcription via recruiting transcription factors, such as NRF2, to the promoter regions of target genes.^[^ ^43^ ^]^ NRF2 activation was reported to reduce CaOx‐induced kidney injury and crystal deposition.^[^ ^44^ ^]^ Our study demonstrated that PGC‐1α activated GPX4 transcription via recruiting NRF2 to the promoter region of GPX4. We also identified a potential NRF2‐binding site in the FTH1 promoter region (989–999 bp upstream of TSS). Interestingly, our animal experiments showed that Sirt1 and PGC‐1α agonists showed a more significant increase in GPX4 expression and protection against CaOx‐induced kidney injury than did the NRF2 agonist, suggesting that Sirt1 and PGC‐1α could protect tubular epithelium in an NRF2‐independent manner. Our previous studies also suggested that Sirt1 inhibits inflammation and oxidative stress, which may be involved in the renoprotective effects of Sirt1.^[^ ^19^ ^]^ In this study, both PGC‐1α and NRF2 agonists partially reversed the downregulation of Sirt1cKO‐induced GPX4 expression and renal tubular injury, which further confirmed that the inhibitory effects of Sirt1 on ferroptosis were PGC‐1α‐ and NRF2‐dependent.

This study has several limitations: While we focused on the roles of ferroptosis, apoptosis, and necroptosis in CaOx nephropathy, other cell death pathways, such as pyroptosis and autophagy, may also regulate CaOx‐induced renal injury, which should be further explored in depth. Our studies concentrated on lipid peroxidation and ROS production changes in tubular epithelial cells, but the alterations in cellular iron metabolism should also be thoroughly investigated. Given that Sirt1 and PGC‐1α also regulated FTH1 and ACSL4, their combined regulatory effects might contribute to their protective role against ferroptosis. Therefore, the underlying molecular mechanisms require further clarification.

Taken together, we demonstrated that CaOx crystals induce ferroptosis in the renal tubular epithelium and identified PTCs as the most severely damaged cell type in mouse kidneys. Sirt1 inhibited CaOx‐induced ferroptosis, kidney injury, and crystal deposition through the PGC‐1α/NRF2/GPX4 pathway. Both Sirt1 and PGC‐1α agonists can be used as effective antagonists of ferroptosis and targets for the treatment of CaOx‐induced kidney injury.

## 4 Experimental Section

### Animal Studies

Renal tubular epithelium‐specific Sirt1 knockout (Sirt1cKO) mice were obtained by crossing Cdh16‐Cre males (T007046, GemPharmatech Inc., China) with Sirt1^fl/fl^ females (T006657, GemPharmatech Inc.) on a C57BL/6J background. The floxed Sirt1 and wild‐type (WT) alleles were assessed by 2 pairs of primers: 5′‐ATCCTGACTTTACCAGCAGTCCAGA‐3′ and 5′‐CCAACTGACCTTGGGCAAGAACAT‐3′ to detect the 5′ arm (353 bp) of the floxed Sirt1 allele; 5′‐GGCATTATGTTAGCAACCAGAGC‐3′ and 5′‐GAGGCGAACCCTAGTCTAAATCAC‐3′ to detect the 3′ arm (282 bp) of the floxed Sirt1 allele. The Cdh16‐Cre sequence was confirmed by the primers 5′‐ GCAGATCTGGCTCTCCAAAG ‐3′ and 5′‐ AGGCAAATTTTGGTGTACGG ‐3,’ which generated a 420 bp product. All animal experiments were performed in compliance with the regulations of the National Institutes of Health Guidance and Ethics Committee of the Zhongnan Hospital of Wuhan University. To establish a mouse model of CaOx‐induced nephrocalcinosis, mice were intraperitoneally injected with GLY (75, 100, and 125 mg kg^−1^/day) for 7 days.^[^ ^45^ ^]^ Fer (5 mg kg^−1^/day), SRT1720 (15 mg kg^−1^/day), EX527 (10 mg kg^−1^/day), ZLN005 (15 mg kg^−1^/day), and TBHQ (10 mg kg^−1^/day) were intraperitoneally injected into mice for 3 days and maintained for the next 7 days of GLY treatment. After 10 days, the blood and kidneys of the mice were collected and used for further analysis. The eyeball blood of the mice was collected for the detection of serum creatinine and BUN using commercial kits (Bioswamp, China) according to the manufacturer's protocol.

### Single Cell Sequencing and Data Analysis

Fresh kidney tissue samples were harvested and cut into small pieces. The kidney tissues were then digested into single‐cell suspensions and added to the loading wells of a 10x Chromium single‐cell instrument (10x Genomics, CA, USA). The cells were bonded to gel beads labeled with barcodes in a droplet. mRNAs marked with unique molecular identifiers (UMIs) captured on gel beads were then incubated with reverse transcriptase to produce full‐length cDNA, which was then amplified for library construction using 10x Chromium Single Cell 3′ Library Kit. A transcript read counts matrix was generated for analysis in R. To control the quality of the dataset, cells with genes per cell of more than 200 and less than 8000, and UMIs per cell of more than 400. and the ratio of mitochondrial UMIs less than 50% were retained for further analysis using the package “Seurat” in R. The highly variable genes were identified using the “FindVariableFeatures” function and used for subsequent dimensionality reduction and unsupervised clustering. To annotate every cell cluster, the “FindAllMarkers” function was used to determine the marker genes across cell clusters, and the CellMarker database, Mouse Cell Atlas, and reported marker genes were applied to identify the cell types. The KEGG database was used for the enrichment analysis of differentially expressed genes in cells between the NC and GLY groups.

### Cell Culture and Lentiviral Transduction

HK‐2 cells were obtained from the China Center for Type Culture Collection (Wuhan, China) and tested to be free of mycoplasma. Cells were maintained in DMEM medium containing 10% fetal bovine serum (Gibco, USA) and cultured in an incubator (Thermo Fisher Scientific, USA) containing 5% CO2 at 37 °C at appropriate humidity. To generate stable cell lines of Sirt1 overexpression and GPX4/PGC‐1α/NRF2 deficiency, relevant lentivirus vectors (pCDH‐Puro and pLKO.1‐Puro) together with psPAX.2 and pMD2.G packaging system were transfected into HEK293T cells using Lipo3000 reagent (Invitrogen, USA) according to the manufacturer's instructions. After 72 h, the viral particles were collected and filtered. HK‐2 cells were then infected and selected with 1 µg mL^−1^ puromycin (Beyotime Biotechnology, China) to obtain stable cell lines. Target sequences of GPX4/PGC‐1α/NRF2 shRNA are presented in Table S2 (Supporting Information).

### Renal CaOx Crystals Detection

The kidney tissues were fixed, embedded, and sectioned. After staining with hematoxylin and eosin (HE), the CaOx crystals were observed under a polarized light optical microscope (Zeiss, Germany). Pizzolato staining was performed to visualize the CaOx crystals under an ordinary microscope. ImageJ software was used to quantify crystal deposition.

### Assessment of Tubular Injury

Kidney sections were stained with periodic acid‐Schiff (PAS) to evaluate tubular injury. Signs of tubular injury such as tubular atrophy or dilation, cast formation, and brush edge loss were evaluated. Scoring was based on the percentage of damaged tubules as described in our previous study.

### Immunohistochemistry (IHC)

For IHC staining, kidney sections were incubated overnight with anti‐GPX4 (1:200, BM5231, Boster, China), anti‐FTH1 (1:200, abs135798, absin, China), anti‐SLC7A11 (1:500, 26864‐1‐AP, proteintech, China), anti‐TFRC (1:1000, 66180‐1‐Ig, proteintech, China), anti‐ACSL4 (1:400, 22401‐1‐AP, proteintech, China), and anti‐4HNE (1:400, ab46545, abcam, USA) at 4 °C. Images of the renal cortex were obtained using a microscope (Olympus, Japan), and ImageJ software was used to quantify the relative expression of these proteins.

### Observation of Mitochondrial Microstructure

After the mice were euthanized, kidney specimens were immediately cut into small pieces and fixed with 2.5% glutaraldehyde. The fixative was washed with phosphate‐buffered saline (PBS), dehydrated, embedded in paraffin, and sectioned. The mitochondrial microstructure was observed using transmission electron microscopy (TEM; FEI, USA). As mitochondrial shrinkage is an important morphological change in ferroptosis, the shape of mitochondria in the tubular epithelium was evaluated by quantifying the aspect ratio (AR). At least three mitochondria were selected for the AR calculations in each field of view.

### Immunofluorescence

HK‐2 cells were seeded onto glass coverslips and cultured for 24 h. After treatment, the medium was removed and the cells were fixed with paraformaldehyde, washed with PBS, and blocked with 3% BSA. Next, the cells were incubated overnight with anti‐4HNE (1:100, ab46545, Abcam, USA) and fluorescently labeled secondary antibodies for 2 h. Kidney sections were incubated overnight with anti‐Sirt1 (1:50, BM3929, Boster, China) and anti‐E‐cadherin (1:100, sc‐8426, SANTA CRUZ, USA) antibodies. Following DAPI staining, images were captured using a fluorescence microscope (Olympus IX71, Japan).

### Quantitative PCR (qPCR)

Total RNA from mouse kidneys and HK‐2 cells was extracted using TRIzol reagent (Invitrogen, USA) and reverse transcribed into cDNA using HiScript III Reverse Transcriptase (Vazyme, China). qPCR was performed using Taq Pro Universal SYBR qPCR Master Mix (Vazyme, China) according to the manufacturer's protocol. The primers used are listed in Table S3 (Supporting Information).

### Immunoblotting

Total protein from mouse kidneys and HK‐2 cells was extracted with RIPA lysis buffer (Servicebio, China), and the protein concentration was measured using the BCA assay (Beyotime Biotechnology, China). The primary antibodies used were as follows: anti‐GPX4 (1:1000, 19 kDa, BM5231, Boster, China), anti‐FTH1 (1:1000, 21 kDa, abs135798, Absin, China), anti‐SLC7A11 (1:1000, 55 kDa, Proteintech, China), anti‐TFRC (1:2000, 90 kDa, 66180‐1‐Ig, Proteintech), anti‐ACSL4 (1:2000, 79 kDa, 22401‐1‐AP, proteintech), and anti‐GAPDH (1:5000, 36 kDa, 60004‐1‐Ig, proteintech, China).

### Chromatin Immunoprecipitation (ChIP):

To verify the binding of PGC‐1α and NRF2 to the promoter regions of GPX4, a ChIP assay was performed with SimpleChIP Enzymatic Chromatin IP Kit (Magnetic Beads, #9003, CST, USA) according to the manufacturer's instruction. Briefly, HK‐2 cells were treated with SRT1720 or ZLN005, fixed, cross‐linked, and fragmented using ultrasound. PGC‐1α (66369‐1‐Ig, proteintech, China) and NRF2 (16396‐1‐AP, proteintech, China) antibodies were used for immunoprecipitation. Finally, ChIP‐qPCR was performed to detect changes in the binding between transcription factors and promoters of GPX4.

### Dual‐Luciferase Reporter Gene Assay

WT and ARE sequence‐mutated (GTGCCACTGCA to TCTCACAGTAC) promoter regions of GPX4 (1 kb upstream of the TSS) were cloned into the luciferase reporter plasmids. Luciferase activity was measured using a Luciferase Assay System (Promega, USA).

### Cell Viability Assay

HK‐2 cells were seeded in 96‐well plates and treated with COM or ferroptosis inducer. The medium was replaced with 10% CCK8 reagent (MCE, USA), followed by incubation for 1 h. Absorbance was measured at 450 nm using a microplate reader (Thermo Fisher Scientific, USA).

### Lipid Peroxidation Assay

After treatment with COM or ferroptosis inducers, HK‐2 cells were incubated with 5 µM BODIPY 581/591 C11 (Thermo Fisher Scientific) for 30 min. Subsequently, HK‐2 cells were washed with PBS and suspended for the detection of lipid peroxidation levels using flow cytometry (BD Bioscience, USA). Data were analyzed using FlowJo V10 software (Tree Star, USA).

### Detection of ROS

To evaluate intracellular ROS production, HK‐2 cells were seeded on coverslips and treated with COM or a ferroptosis inducer. The cells were then incubated with DCFH‐DA (Beyotime) for 20 min in the dark. In addition, to measure the renal level of ROS, mice's kidney was embedded into an OCT compound and frozen at −80 °C. Frozen kidney tissues were sectioned and stained with dihydroethidium (DHE) for 30 min. After washing with PBS, the HK‐2 cells and kidney sections were observed under a fluorescence microscope (Olympus, Japan).

### MDA Assay

The MDA concentration in HK‐2 cells and kidney tissue lysates was determined using a Lipid Peroxidation Assay Kit (Beyotime, China). Briefly, the test samples and MDA solutions were mixed and heated for 15 min. After centrifugation, the supernatants were transferred to 96‐well plates and the absorbance at 532 nm was measured using a microplate reader (Thermo Fisher Scientific, USA).

### Statistics

Statistical analysis of the single‐cell sequencing data was performed in R using the by utilizing “Seurat” package. All experiments were repeated at least in triplicates, and data were presented as mean ± standard deviation (SD). All the statistical analyses employed in this study are illustrated in the related figure legends. Two‐tailed Student's t‐tests and one‐way or two‐way analysis of variance (ANOVA) were used to analyze the data, and P values were calculated using GraphPad Prism software (version 8.0). *p* < 0.05 was considered significant (ns: not significant, ^*^ *p* < 0.05, ^**^ *p* < 0.01, ^***^ *p* < 0.001, ^****^ *p* < 0.0001).

All data required to evaluate the conclusions of this study are presented in the paper and/or Supporting Information. The single‐cell sequencing data generated in this study were deposited in GEO (GSE 269465).

## Conflict of Interest

The authors declare no conflict of interest.

## Author Contributions

C.D. and B.L. contributed equally to this work. All authors contributed to this work and approved the submitted version. C.D., B.L., Y.G. and H.X. conceived the project and designed the study. C.D., B.L., H.R.L., Y.J.Z., X.Y.Y. and K.L. performed the experiments. X.L.W., X.M.M., H.H.W., Z.Z.X. and Y.H.Z. analyze the data. C.D. and B.L. wrote the manuscript. Z.Q.H., Y.G. and H.X. improved the manuscript.

## Supporting information



Supporting Information

## Data Availability

The data that support the findings of this study are openly available in GEO at https://www.ncbi.nlm.nih.gov/geo/, reference number 269465.

## References

[1] S. R. Mulay , C. Shi , X. Ma , H. J. Anders , Kidney Dis (Basel) 2018, 4, 49. 29998119 10.1159/000487671 PMC6029228

[2] B. S. Franklin , M. S. Mangan , E. Latz , Annu. Rev. Immunol. 2016, 34, 173. 26772211 10.1146/annurev-immunol-041015-055539

[3] S. R. Khan , M. S. Pearle , W. G. Robertson , G. Gambaro , B. K. Canales , S. Doizi , O. Traxer , H. G. Tiselius , Nat. Rev. Dis. Primers 2016, 2, 16008. 27188687 10.1038/nrdp.2016.8 PMC5685519

[4] K. Newton , A. Strasser , N. Kayagaki , V. M. Dixit , Cell 2024, 187, 235. 38242081 10.1016/j.cell.2023.11.044

[5] S. J. Dixon , K. M. Lemberg , M. R. Lamprecht , R. Skouta , E. M. Zaitsev , C. E. Gleason , D. N. Patel , A. J. Bauer , A. M. Cantley , W. S. Yang , B. Morrison 3rd , B. R. Stockwell , Cell 2012, 149, 1060. 22632970 10.1016/j.cell.2012.03.042 PMC3367386

[6] P. Tsvetkov , S. Coy , B. Petrova , M. Dreishpoon , A. Verma , M. Abdusamad , J. Rossen , L. Joesch‐Cohen , R. Humeidi , R. D. Spangler , J. K. Eaton , E. Frenkel , M. Kocak , S. M. Corsello , S. Lutsenko , N. Kanarek , S. Santagata , T. R. Golub , Science 2022, 375, 1254. 35298263 10.1126/science.abf0529 PMC9273333

[7] S. R. Mulay , J. Desai , S. V. Kumar , J. N. Eberhard , D. Thomasova , S. Romoli , M. Grigorescu , O. P. Kulkarni , B. Popper , V. Vielhauer , G. Zuchtriegel , C. Reichel , J. H. Brasen , P. Romagnani , R. Bilyy , L. E. Munoz , M. Herrmann , H. Liapis , S. Krautwald , A. Linkermann , H. J. Anders , Nat. Commun. 2016, 7, 10274. 26817517 10.1038/ncomms10274 PMC4738349

[8] S. R. Mulay , M. M. Honarpisheh , O. Foresto‐Neto , C. Shi , J. Desai , Z. B. Zhao , J. A. Marschner , B. Popper , E. M. Buhl , P. Boor , A. Linkermann , H. Liapis , R. Bilyy , M. Herrmann , P. Romagnani , I. Belevich , E. Jokitalo , J. U. Becker , H. J. Anders , J. Am. Soc. Nephrol. 2019, 30, 1857. 31296606 10.1681/ASN.2018121218 PMC6779355

[9] Q. Song , W. Liao , X. Chen , Z. He , D. Li , B. Li , J. Liu , L. Liu , Y. Xiong , C. Song , S. Yang , Oxid. Med. Cell. Longev. 2021, 2021, 6630343. 34659638 10.1155/2021/6630343 PMC8514920

[10] B. R. Stockwell , J. P. Friedmann Angeli , H. Bayir , A. I. Bush , M. Conrad , S. J. Dixon , S. Fulda , S. Gascon , S. K. Hatzios , V. E. Kagan , K. Noel , X. Jiang , A. Linkermann , M. E. Murphy , M. Overholtzer , A. Oyagi , G. C. Pagnussat , J. Park , Q. Ran , C. S. Rosenfeld , K. Salnikow , D. Tang , F. M. Torti , S. V. Torti , S. Toyokuni , K. A. Woerpel , D. D. Zhang , Cell 2017, 171, 273. 28985560 10.1016/j.cell.2017.09.021 PMC5685180

[11] W. S. Yang , R. SriRamaratnam , M. E. Welsch , K. Shimada , R. Skouta , V. S. Viswanathan , J. H. Cheah , P. A. Clemons , A. F. Shamji , C. B. Clish , L. M. Brown , A. W. Girotti , V. W. Cornish , S. L. Schreiber , B. R. Stockwell , Cell 2014, 156, 317. 24439385 10.1016/j.cell.2013.12.010 PMC4076414

[12] L. Guarente , Genes Dev. 2013, 27, 2072. 24115767 10.1101/gad.227439.113 PMC3850092

[13] M. Morigi , L. Perico , A. Benigni , J. Am. Soc. Nephrol. 2018, 29, 1799. 29712732 10.1681/ASN.2017111218 PMC6050939

[14] A. D. Karikkakkavil Prakashan , S. P. Muthukumar , A. Martin , Mol. Nutr. Food Res. 2024, 68, 2200660. 10.1002/mnfr.202200660 38549461

[15] S. Shen , M. Shen , L. Kuang , K. Yang , S. Wu , X. Liu , Y. Wang , Y. Wang , Pharmacol. Res. 2024, 199, 107037. 38070792 10.1016/j.phrs.2023.107037

[16] Y. Zhang , T. Li , M. Pan , W. Wang , W. Huang , Y. Yuan , Z. Xie , Y. Chen , J. Peng , X. Li , Y. Meng , J. Transl. Med. 2022, 20, 222. 35568871 10.1186/s12967-022-03408-5 PMC9107262

[17] Y.‐J. Tang , Z. Zhang , T. Yan , K. Chen , G.‐F. Xu , S.‐Q. Xiong , D.‐Q. Wu , J. Chen , P. A. Jose , C.‐Y. Zeng , J.‐J. Fu , Cardiovasc. Diabetol. 2024, 23, 116. 38566123 10.1186/s12933-024-02183-5 PMC10985893

[18] Q. K. Lv , K. X. Tao , X. Y. Yao , M. Z. Pang , B. E. Cao , C. F. Liu , F. Wang , J. Pineal Res. 2024, 76, e12948. 38488331 10.1111/jpi.12948

[19] H. Liu , C. Duan , X. Yang , J. Liu , Y. Deng , H. G. Tiselius , Z. Ye , T. Wang , J. Xing , H. Xu , Signal Transduct Target Ther 2023, 8, 38. 36702833 10.1038/s41392-022-01232-3 PMC9879973

[20] P. S. Rakshe , B. J. Dutta , S. Chib , N. Maurya , S. Singh , Ageing Res. Rev. 2024, 96, 102255. 38490497 10.1016/j.arr.2024.102255

[21] K. Ye , Y. Zhao , W. Huang , Y. Zhu , Sci. Rep. 2024, 14, 17867. 39090182 10.1038/s41598-024-68227-8 PMC11294604

[22] X. Su , Q. Li , M. Yang , W. Zhang , X. Liu , Y. Ba , Q. Deng , Y. Zhang , L. Han , H. Huang , Exp. Neurol. 2024, 380, 114899. 39059737 10.1016/j.expneurol.2024.114899

[23] J. St‐Pierre , S. Drori , M. Uldry , J. M. Silvaggi , J. Rhee , S. Jager , C. Handschin , K. Zheng , J. Lin , W. Yang , D. K. Simon , R. Bachoo , B. M. Spiegelman , Cell 2006, 127, 397. 17055439 10.1016/j.cell.2006.09.024

[24] S. Jamwal , J. K. Blackburn , J. D. Elsworth , Pharmacol. Ther. 2021, 219, 107705. 33039420 10.1016/j.pharmthera.2020.107705 PMC7887032

[25] M. R. Lynch , M. T. Tran , K. M. Ralto , Z. K. Zsengeller , V. Raman , S. S. Bhasin , N. Sun , X. Chen , D. Brown Rovira II , K. Taguchi , C. R. Brooks , I. E. Stillman , M. K. Bhasin , T. Finkel , S. M. Parikh , JCI Insight 2019, 5, 126749. 30870143 10.1172/jci.insight.126749 PMC6538327

[26] P. Dhillon , J. Park , C. Hurtado Del Pozo , L. Li , T. Doke , S. Huang , J. Zhao , H. M. Kang , R. Shrestra , M. S. Balzer , S. Chatterjee , P. Prado , S. Y. Han , H. Liu , X. Sheng , P. Dierickx , K. Batmanov , J. P. Romero , F. Prosper , M. Li , L. Pei , J. Kim , N. Montserrat , K. Susztak , Cell Metab. 2021, 33, 379. 33301705 10.1016/j.cmet.2020.11.011 PMC9259369

[27] W. S. Yang , B. R. Stockwell , Chem. Biol. 2008, 15, 234. 18355723 10.1016/j.chembiol.2008.02.010 PMC2683762

[28] M. Salman , H. Tabassum , S. Parvez , Mol. Neurobiol. 2020, 57, 2870. 32399817 10.1007/s12035-020-01924-3

[29] C. Xu , S. Ni , N. Xu , G. Yin , Y. Yu , B. Zhou , G. Zhao , L. Wang , R. Zhu , S. Jiang , Y. Wang , Oxid. Med. Cell. Longev. 2022, 2022, 3531995. 36439689 10.1155/2022/3531995 PMC9691334

[30] H. Sun , H. Cai , C. Xu , H. Zhai , F. Lux , Y. Xie , L. Feng , L. Du , Y. Liu , X. Sun , Q. Wang , H. Song , N. He , M. Zhang , K. Ji , J. Wang , Y. Gu , G. Leduc , T. Doussineau , Y. Wang , Q. Liu , O. Tillement , J Nanobiotechnology 2022, 20, 449. 36242003 10.1186/s12951-022-01654-9 PMC9569109

[31] S. R. Mulay , J. N. Eberhard , V. Pfann , J. A. Marschner , M. N. Darisipudi , C. Daniel , S. Romoli , J. Desai , M. Grigorescu , S. V. Kumar , B. Rathkolb , E. Wolf , M. Hrabe de Angelis , T. Bauerle , B. Dietel , C. A. Wagner , K. Amann , K. U. Eckardt , P. S. Aronson , H. J. Anders , F. Knauf , Am. J. Physiol. Renal Physiol. 2016, 310, F785. 26764204 10.1152/ajprenal.00488.2015 PMC5504458

[32] H. Liu , X. Yang , K. Tang , T. Ye , C. Duan , P. Lv , L. Yan , X. Wu , Z. Chen , J. Liu , Y. Deng , G. Zeng , J. Xing , Z. Ye , H. Xu , Theranostics 2020, 10, 7319. 32641994 10.7150/thno.44054 PMC7330860

[33] T. Ding , T. Zhao , Y. Li , Z. Liu , J. Ding , B. Ji , Y. Wang , Z. Guo , Phytomedicine 2021, 86, 153562. 33857849 10.1016/j.phymed.2021.153562

[34] J. Liu , K. Yang , Y. Jin , Y. Liu , Y. Chen , X. Zhang , S. Yu , E. Song , S. Chen , J. Zhang , G. Jing , R. An , Cell Prolif 2020, 53, e12902. 32945585 10.1111/cpr.12902 PMC7574868

[35] F. Dong , S. Jiang , C. Tang , X. Wang , X. Ren , Q. Wei , J. Tian , W. Hu , J. Guo , X. Fu , L. Liu , A. Patzak , P. B. Persson , F. Gao , E. Y. Lai , L. Zhao , F. Radic. Biol. Med. 2022, 179, 288. 10.1016/j.freeradbiomed.2021.11.010 34767921

[36] X. Duan , Z. Kong , X. Mai , Y. Lan , Y. Liu , Z. Yang , Z. Zhao , T. Deng , T. Zeng , C. Cai , S. Li , W. Zhong , W. Wu , G. Zeng , Redox Biol. 2018, 16, 414. 29653411 10.1016/j.redox.2018.03.019 PMC5953241

[37] Y. Zou , M. J. Palte , A. A. Deik , H. Li , J. K. Eaton , W. Wang , Y. Y. Tseng , R. Deasy , M. Kost‐Alimova , V. Dancik , E. S. Leshchiner , V. S. Viswanathan , S. Signoretti , T. K. Choueiri , J. S. Boehm , B. K. Wagner , J. G. Doench , C. B. Clish , P. A. Clemons , S. L. Schreiber , Nat. Commun. 2019, 10, 1617. 30962421 10.1038/s41467-019-09277-9 PMC6453886

[38] Y. Ding , X. Chen , C. Liu , W. Ge , Q. Wang , X. Hao , M. Wang , Y. Chen , Q. Zhang , J. Hematol. Oncol. 2021, 14, 19. 33472669 10.1186/s13045-020-01016-8 PMC7816340

[39] Y. Tian , J. Lu , X. Hao , H. Li , G. Zhang , X. Liu , X. Li , C. Zhao , W. Kuang , D. Chen , M. Zhu , Neurotherapeutics 2020, 17, 1796. 32959272 10.1007/s13311-020-00929-z PMC7851296

[40] N. Kong , X. Chen , J. Feng , T. Duan , S. Liu , X. Sun , P. Chen , T. Pan , L. Yan , T. Jin , Y. Xiang , Q. Gao , C. Wen , W. Ma , W. Liu , M. Zhang , Z. Yang , W. Wang , R. Zhang , B. Chen , T. Xie , X. Sui , W. Tao , Acta Pharm. Sin. B. 2021, 11, 4045. 35024325 10.1016/j.apsb.2021.03.036 PMC8727776

[41] P. Liao , W. Wang , W. Wang , I. Kryczek , X. Li , Y. Bian , A. Sell , S. Wei , S. Grove , J. K. Johnson , P. D. Kennedy , M. Gijon , Y. M. Shah , W. Zou , Cancer Cell 2022, 40, 365. 35216678 10.1016/j.ccell.2022.02.003 PMC9007863

[42] Q. Z. Tuo , Y. Liu , Z. Xiang , H. F. Yan , T. Zou , Y. Shu , X. L. Ding , J. J. Zou , S. Xu , F. Tang , Y. Q. Gong , X. L. Li , Y. J. Guo , Z. Y. Zheng , A. P. Deng , Z. Z. Yang , W. J. Li , S. T. Zhang , S. Ayton , A. I. Bush , H. Xu , L. Dai , B. Dong , P. Lei , Signal Transduct Target Ther 2022, 7, 59. 35197442 10.1038/s41392-022-00917-z PMC8866433

[43] P. Abrescia , L. Treppiccione , M. Rossi , P. Bergamo , Prog. Lipid Res. 2020, 80, 101066. 32979455 10.1016/j.plipres.2020.101066

[44] H. Lu , X. Sun , M. Jia , F. Sun , J. Zhu , X. Chen , K. Chen , K. Jiang , Oxid. Med. Cell. Longev. 2021, 2021, 5527137. 34691355 10.1155/2021/5527137 PMC8531781

[45] A. Okada , S. Nomura , Y. Higashibata , M. Hirose , B. Gao , M. Yoshimura , Y. Itoh , T. Yasui , K. Tozawa , K. Kohri , Urol. Res. 2007, 35, 89. 17393196 10.1007/s00240-007-0082-8

