# Supplementary material for: Sirtuin1 Suppresses Calcium Oxalate Nephropathy via Inhibition of Renal Proximal Tubular Cell Ferroptosis Through PGC‐1α‐mediated Transcriptional Coactivation
Source: Adv Sci (Weinh). 2024 Nov 5;11(48):2408945. doi: 10.1002/advs.202408945 (PMC11672264; doi:10.1002/advs.202408945)
Supplement: Supplementary file 1 — Supporting Information [file ADVS-11-2408945-s001.docx]

**Sirtuin1 Suppresses Calcium Oxalate Nephropathy via Inhibiting Renal Proximal Tubular Cell Ferroptosis through PGC-1α-mediated Transcriptional Coactivation**

**Chen Duan^✝^, Bo Li^✝^, Haoran Liu, Yangjun Zhang, Xiangyang Yao, Kai Liu, Xiaoliang Wu, Xiongmin Mao, Huahui Wu, Zhenzhen Xu, Yahua Zhong, Zhiquan Hu, Yan Gong^*^, and Hua Xu ^*^**

***Correspondence:**

Hua Xu, xu-hua@whu.edu.cn
Yan Gong, yan.gong@whu.edu.cn

^✝^These authors contributed equally to this work.

**Supplementary Figures**

**Fig. S1**

**
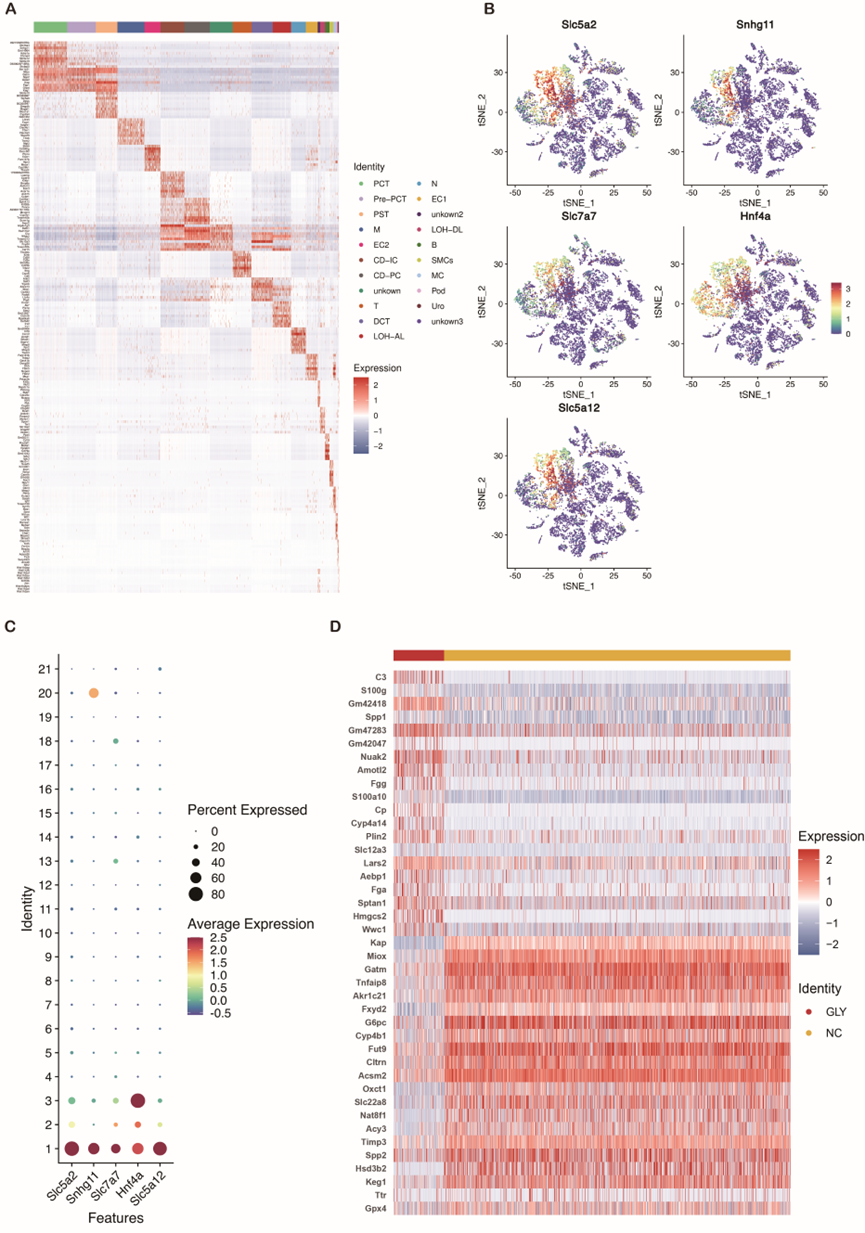
**

**Fig S1. Expression of cell type markers across different clusters.**

(**A**) Scaled gene expression of marker genes in 21 clusters. (**B, C**) Scaled gene expression of PCT marker genes (Slc5a2, Snhg11, Slc7a7, Hnf4a, Slc5a12) in 21 clusters. (**D**) DEGs in PCT cells between NC and GLY groups. Detailed statistics were presented in Methods.

**Fig. S2**

**
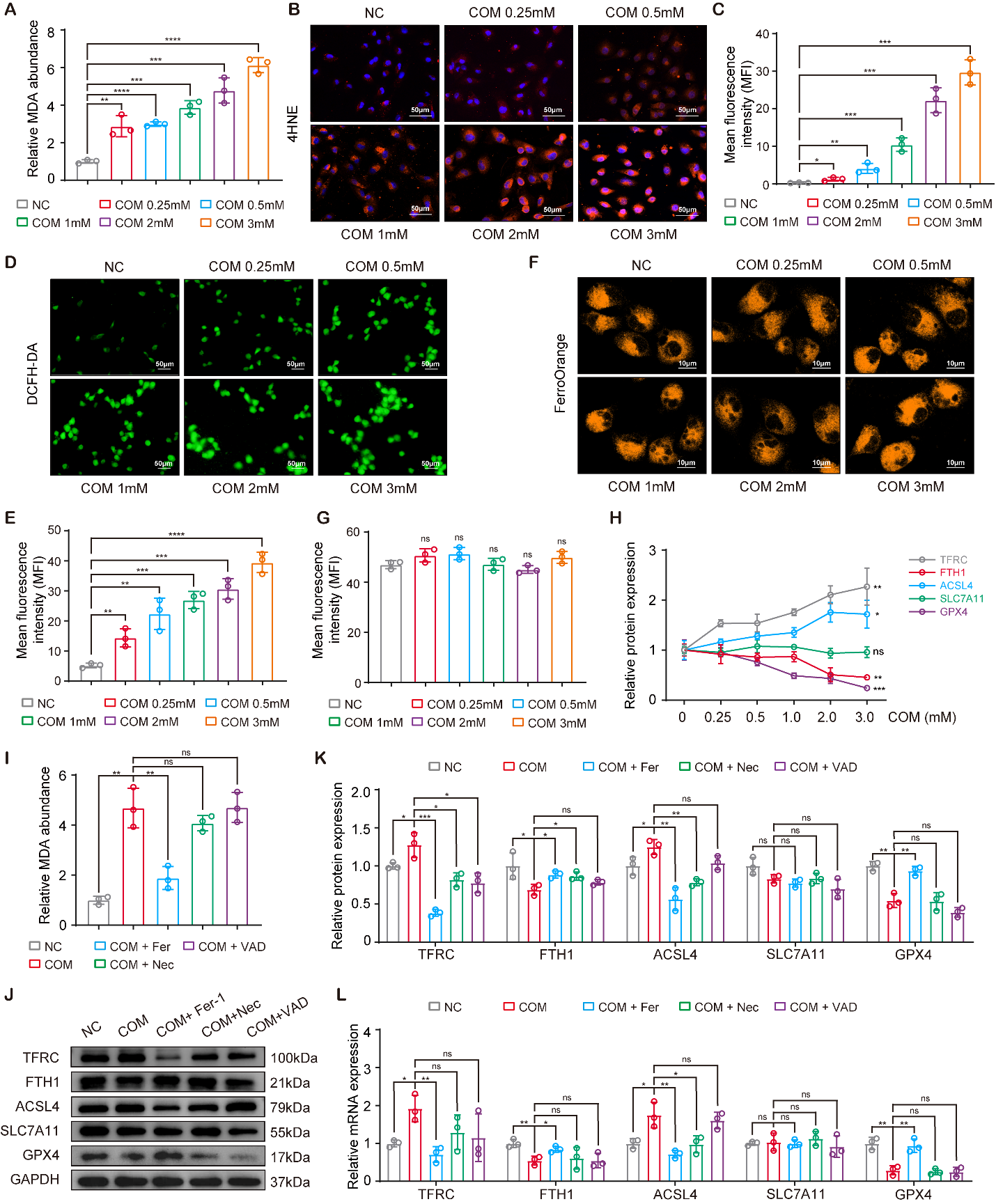
**

**Fig S2. CaOx crystals induced ferroptosis in HK-2 cells.**

(**A**) Level of MDA was measured in COM-treated HK-2 cells. (**B, C**) Level of 4HNE was detected by immunofluorescence. (**D, E**) Intracellular ROS generation was detected by DCFH-DA staining. (**F, G**) Level of Fe^2+^ was detected by FerroOrange fluorescence probe. (**H**) Western blot quantification of TFRC, FTH1, ACSL4, SLC7A11 and GPX4 expression in COM-treated HK-2 cells. (**I**) Level of MDA was measured in HK-2 cells treated with COM and Fer/Nec/VAD. Western blot (**J, K**) and qPCR (**L**) revealed the expression of TFRC, FTH1, ACSL4, SLC7A11 and GPX4 in HK-2 cells treated with COM and Fer/Nec/VAD. Data were presented as mean ± SD, n = 3, and P value was determined by one-way ANOVA (**A, C, E, G-I, K, L**). * *P* < 0.05, ** *P* < 0.01, *** *P* < 0.001, **** *P* < 0.0001.

**Fig. S3**


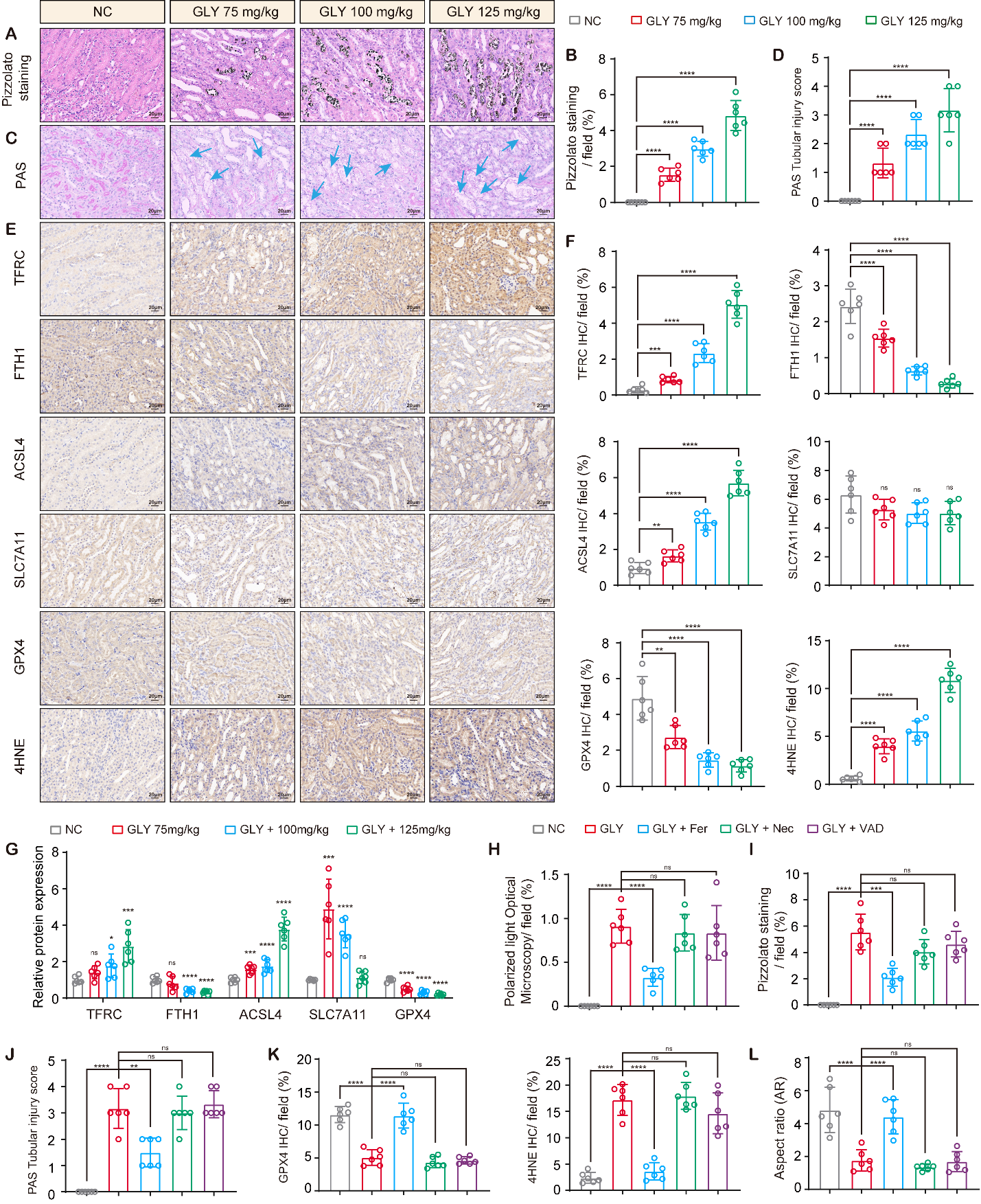


**Fig S3. Inhibition of ferroptosis reduced glyoxylate-induced tubular injury and CaOx crystal deposition.**

C57/B6J mice were intraperitoneally injected with GLY (75 mg/kg, 100 mg/kg, and 125 mg/kg) to establish a CaOx nephrocalcinosis model. The deposition of CaOx was evaluated by Pizzolato staining (**A, B**). (**C, D**) PAS staining was used for the scoring of tubular injury. The blue arrows highlight damaged renal tubules. (**E, F**) Immunohistochemical staining showed the expression of TFRC, FTH1, ACSL4, SLC7A11, GPX4 and 4HNE in mice kidney. Mice were pretreated with Fer (1 mg/kg/d), Nec (1 mg/kg/d) or VAD (6 mg/kg/d) for 3 days and then establish a mouse model of CaOx nephrocalcinosis (100 mg/kg/d). (**G**) Western blot quantification of TFRC, FTH1, ACSL4, SLC7A11 and GPX4 expression in mouse kidney. (**H, I**) The ratio of the areas of kidneys with crystal deposition, as detected by polarized light optical microscopy and Pizzolato staining. (**J**) Scoring of tubular injury. (**K**) The ratio of the areas with positive expression of GPX4 and 4HNE in renal tissue, as determined by IHC. (**L**) Quantification of mitochondrial shape transitions by aspect ratio. Data were presented as mean ± SD, n = 6, and P value was determined by one-way ANOVA (**B, D, F-L**). * *P* < 0.05, ** *P* < 0.01, *** *P* < 0.001, **** *P* < 0.0001.

**Fig. S4**


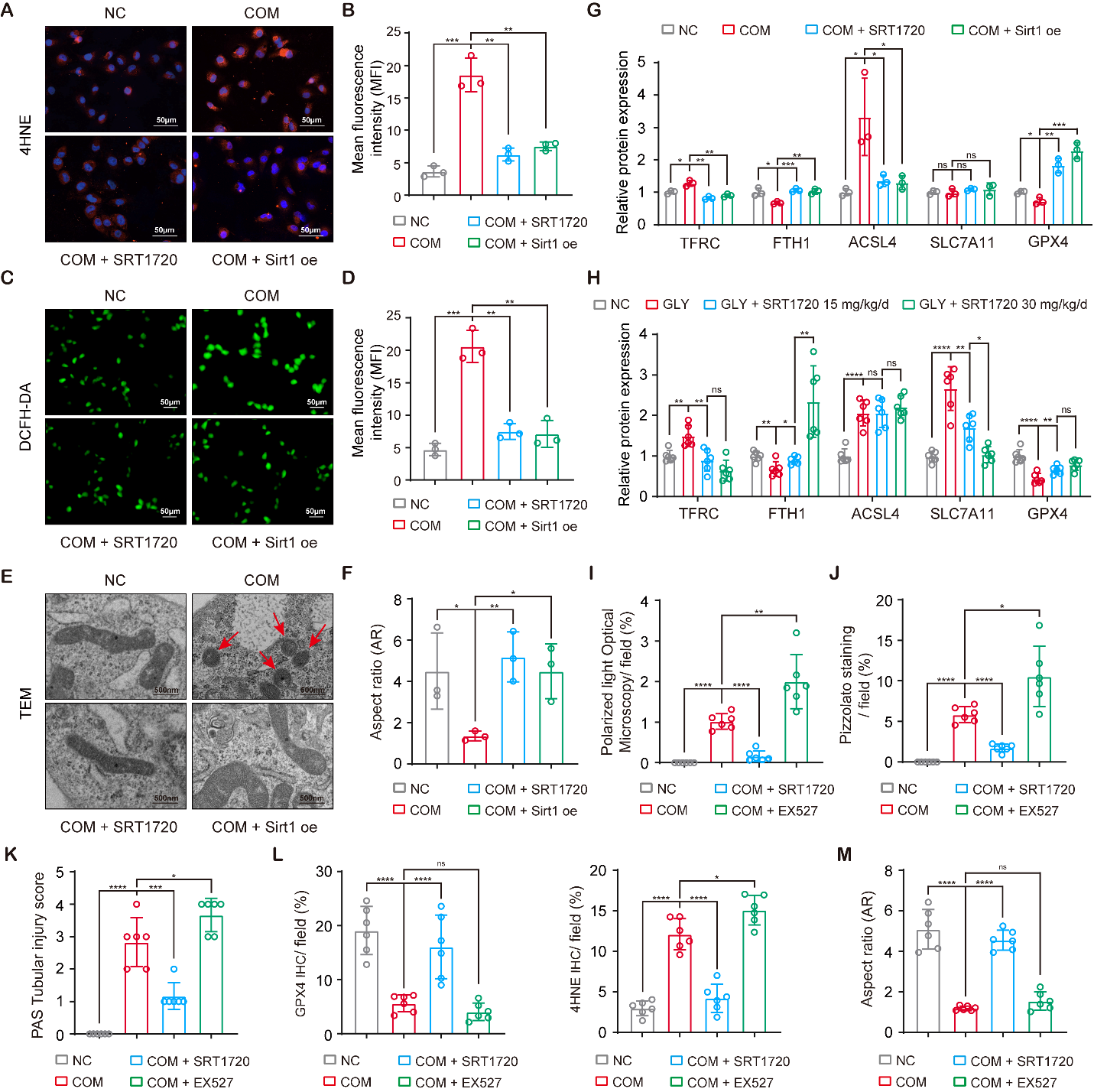


**Fig S4. Sirt1 inhibited CaOx-induced ferroptosis in tubular epithelium.**

(**A, B**) Level of 4HNE was detected by immunofluorescence. (**C, D**) Intracellular ROS generation was detected by DCFH-DA staining. (**E**) The mitochondrial microstructure was observed under a TEM. The red arrows indicate the morphologically abnormal mitochondria, characterized by mitochondrial shrinkage and disorganized cristae. (**F**) Quantification of mitochondrial shape transitions by aspect ratio. (**G**) Western blot quantification of ferroptosis associated markers in HK-2 cells. (**H**) Western blot quantification of ferroptosis associated markers in mice kidney. (**I, J**) The ratio of the areas of kidneys with crystal deposition, as detected by polarized light optical microscopy and Pizzolato staining. (**K**) Scoring of tubular injury. (**L**) The ratio of the areas with positive expression of GPX4 and 4HNE in renal tissue, as determined by IHC. (**M**) Quantification of mitochondrial shape transitions by aspect ratio. Data were presented as mean ± SD, n = 3 in vitro and n = 6 in vivo, and P value was determined by one-way ANOVA (**B, D, F-M**). * *P* < 0.05, ** *P* < 0.01, *** *P* < 0.001, **** *P* < 0.0001.

**Fig. S5**


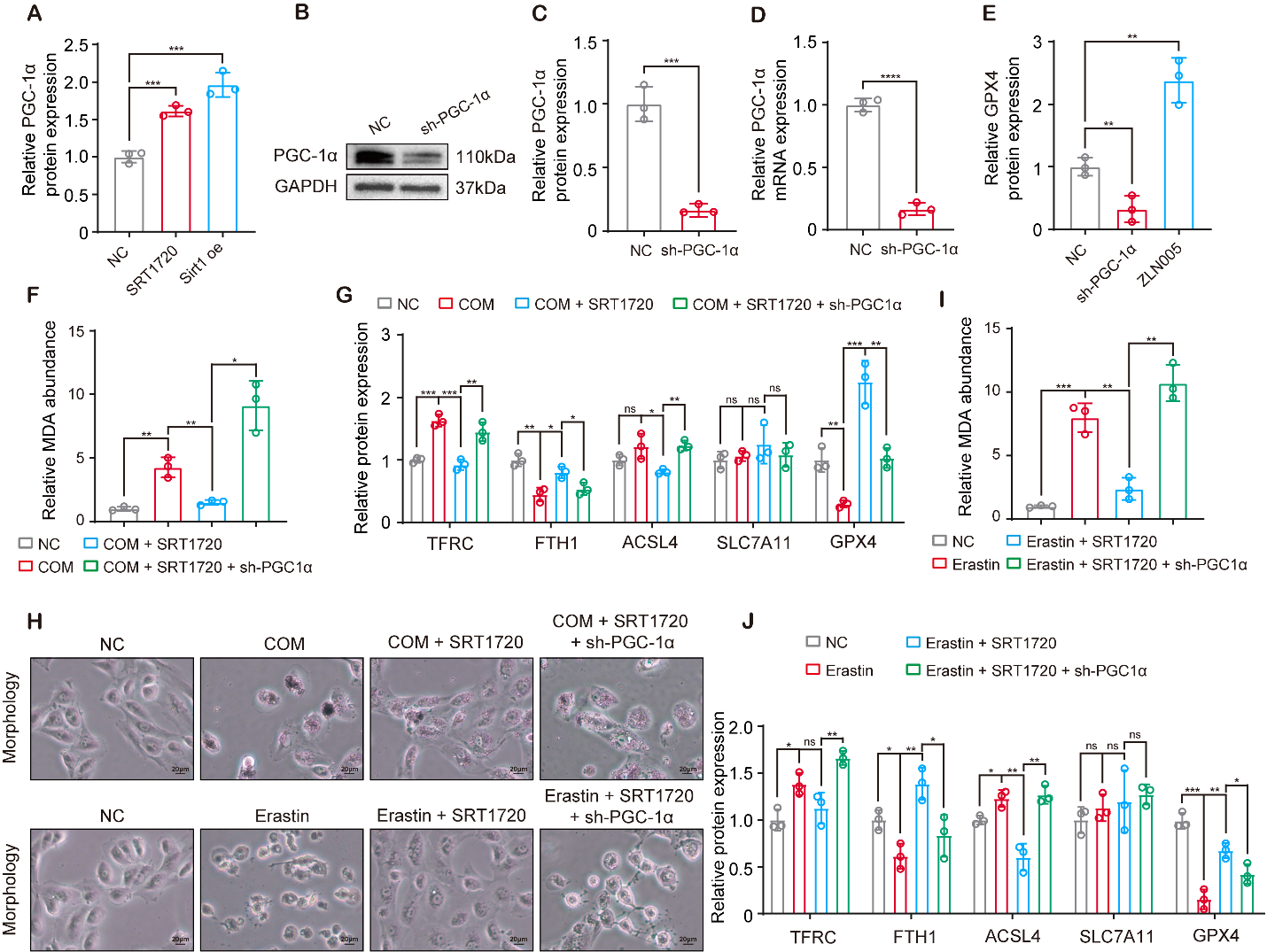


**Fig S5. Sirt1 inhibited ferroptosis in tubular epithelium via a PGC-1α/GPX4 pathway.**

(**A**) Western blot quantification of PGC-1α in Sirt1 activated and overexpressed HK-2 cells. (**B-D**) The level of protein and mRNA expression of PGC-1α in Sh^ctrl^ and PGC-1α^sh^ HK-2 cells. (**E**) Western blot quantification of GPX4 in Sh^ctrl^ and PGC-1α^sh^ HK-2 cells treated by ZLN005. (**F**) Level of MDA in Sh^ctrl^ and PGC-1α^sh^ HK-2 cells treated by COM and SRT1720. (**G**) Western blot quantification of ferroptosis associated markers in Sh^ctrl^ and PGC-1α^sh^ HK-2 cells treated by COM and SRT1720. (**H**) Morphology of HK-2 was observed under a microscope. (**I**) Level of MDA in Sh^ctrl^ and PGC-1α^sh^ HK-2 cells treated by erastin and SRT1720. (**J**) Western blot quantification of ferroptosis associated markers in Sh^ctrl^ and PGC-1α^sh^ HK-2 cells treated by erastin and SRT1720. Data were presented as mean ± SD, n = 3, and P value was determined by one-way ANOVA (**A, C-G, I, J**). * *P* < 0.05, ** *P* < 0.01, *** *P* < 0.001, **** *P* < 0.0001.

**Fig. S6**


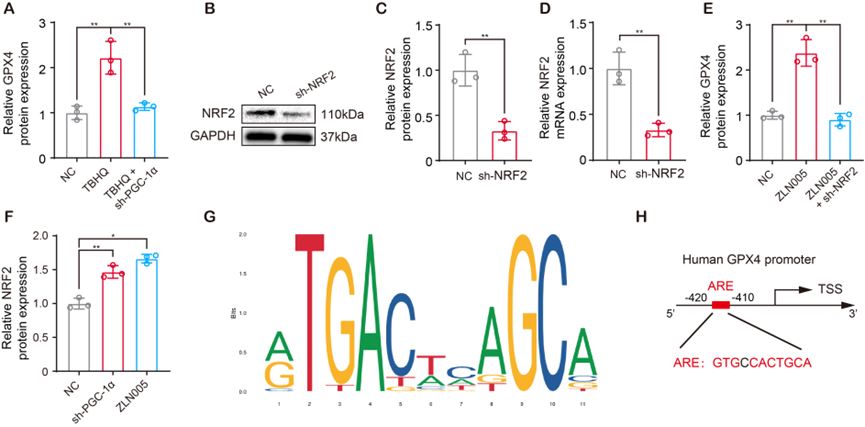


**Fig S6. PGC-1α and NRF2 coactivated GPX4 transcription.**

(**A**) Western blot quantification of GPX4 in Sh^ctrl^ and PGC-1α^sh^ HK-2 cells treated by TBHQ. (**B-D**) The level of protein and mRNA expression of NRF2 in Sh^ctrl^ and NRF2^sh^ HK-2 cells. (**E**) Western blot quantification of GPX4 in Sh^ctrl^ and NRF2^sh^ HK-2 cells treated by TBHQ. (**F**) Western blot quantification of NRF2 in Sh^ctrl^ and PGC-1α^sh^ HK-2 cells treated by ZLN005. (**G**) NRF2 binds to the AREs sequence in gene promoter. (**H**) The potential binding site of NRF2 in GPX4 promoter. Data were presented as mean ± SD, n = 3, and P value was determined by one-way ANOVA (**A, C-F**). * *P* < 0.05, ** *P* < 0.01, *** *P* < 0.001, **** *P* < 0.0001.

**Fig. S7**


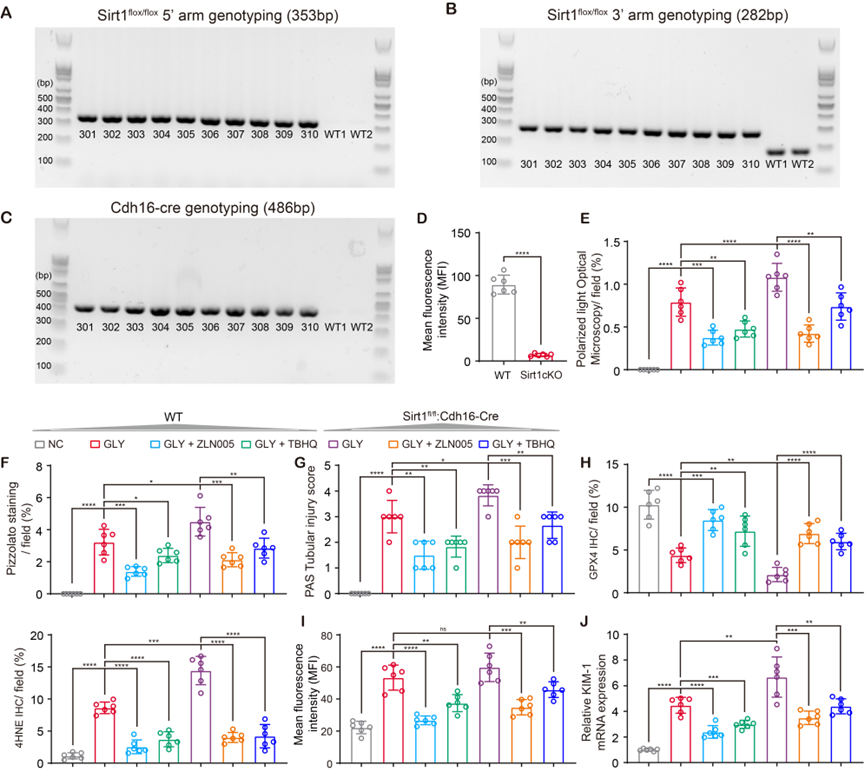


**Fig S7. Sirt1 inhibited CaOx-induced ferroptosis, crystal deposition and kidney injury through PGC-1α/NRF2 signaling.**

(**A-C**) The sequences of Sirt1-LoxP and Cdh16-cre were detected by PCR. 100bp DNA Ladder was used as the marker. (**D**) Level of Sirt1 was detected by immunofluorescence. (**E, F**) The ratio of the areas of kidneys with crystal deposition, as detected by polarized light optical microscopy and Pizzolato staining. (**G**) PAS staining was used for the scoring of tubular injury. (**H**) The ratio of the areas with positive expression of GPX4 and 4HNE in renal tissue, as determined by IHC. (**I**) Kidney ROS production was determined by DHE staining. (**J**) The expression of KIM-1 mRNA in tissues of mice kidney was determined by qPCR. Data were presented as mean ± SD, n = 6, and P value was determined by one-way ANOVA (**D-J**). * P < 0.05, ** P < 0.01, *** P < 0.001, **** P < 0.0001.

**Supplementary Tables**

**Table S1. Cell cluster names**

| **Clusters** | **Label** | **Annotation** |
| --- | --- | --- |
| 1 | PCT | proximal convoluted tubule |
| 2 | Pre-PCT | Pre-proximal convoluted tubule |
| 3 | PST | proximal straight tubule |
| 4 | M | Macrophage |
| 5 | EC2 | endothelial cell 2 |
| 6 | CD-IC | collecting duct-intercalated cell |
| 7 | CD-PC | collecting duct-principal cell |
| 8 | unknown 1 | unknown 1 |
| 9 | T | T cell |
| 10 | DCT | distal convoluted tubule |
| 11 | LOH-AL | ascending limb of loop of Henle |
| 12 | N | Neutrophi |
| 13 | EC1 | endothelial cell 1 |
| 14 | unknown 2 | unknown 2 |
| 15 | LOH-DL | descending limb of loop of Henle |
| 16 | B | B cell |
| 17 | SMCs | smooth muscle cells |
| 18 | MC | mesangial cell |
| 19 | Pod | podocyte |
| 20 | Uro | urothelium |
| 21 | unknown 3 | unknown 3 |

**Table S2. Oligos sequences used in shRNA vectors**

| **Name** | **TARGET SEQUENCE (5'-3')** |
| --- | --- |
| GPX4-shRNA-1 | GTGAGGCAAGACCGAAGTAAA |
| GPX4-shRNA-2 | CAAATTCGATATGTTCAGCAA |
| GPX4-shRNA-3 | GCACATGGTTAACCTGGACAA |
| GPX4-shRNA-4 | GTGGATGAAGATCCAACCCAA |
| PGC-1α-shRNA-1 | CCTCCTCATAAAGCCAACCAA |
| PGC-1α-shRNA-2 | CGACTTGGATACAGACAGCTT |
| PGC-1α-shRNA-3 | GACAGCGAAGATGAAAGTGAT |
| PGC-1α-shRNA-4 | TATGACAGCTACGAGGAATAT |
| Nrf2-shRNA-1 | CCGGCATTTCACTAAACACAA |
| Nrf2-shRNA-2 | CTTGCATTAATTCGGGATATA |
| Nrf2-shRNA-3 | AGAGCAAGATTTAGATCATTT |
| Nrf2-shRNA-4 | GCTCCTACTGTGATGTGAAAT |

**Table S3. List of primer sequences used for real-time qPCR analysis**

| **Primer** | **Name** | **SPECIES** | **(5'-3')** | **SEQUENCE (5'-3')** |
| --- | --- | --- | --- | --- |
| qRT-PCR | GPX4 | human | Forward | AGGAGCCAGGGAGTAACGAA |
|  |  | human | Reverse | CCACTTGATGGCATTTCCCAG |
|  | Gpx4 | mouse | Forward | AAAGTCCTAGGAAACGCCCG |
|  |  | mouse | Reverse | GCACACGAAACCCCTGTACT |
|  | TFRC | human | Forward | TCGTGTCATGAGAGTGGAGT |
|  |  | human | Reverse | CCCCAGAAGACATGTCGGAA |
|  | Tfrc | mouse | Forward | TCACACTCTCTCAGCTTTAGTG |
|  |  | mouse | Reverse | TGGTTTCTGAAGAGGGTTTCAT |
|  | FTH1 | human | Forward | AGTGGGAGTTGCTGTTGAAGTCG |
|  |  | human | Reverse | AGTGGGAGTTGCTGTTGAAGTCG |
|  | Fth1 | mouse | Forward | TAAAGAAACCAGACCGTGATGA |
|  |  | mouse | Reverse | ATTCACACTCTTTTCCAAGTGC |
|  | SLC7A11 | human | Forward | ATCGTCCTTTCAAGGTGCCACT |
|  |  | human | Reverse | CCCTATTTTGTGTCTCCCCTTGG |
|  | Slc7a11 | mouse | Forward | CTATTTTACCACCATCAGTGCG |
|  |  | mouse | Reverse | ATCGGGACTGCTAATGAGAATT |
|  | ACSL4 | human | Forward | ATACCTGGACTGGGACCGAA |
|  |  | human | Reverse | TCCGGAACAGCAGCCATAAG |
|  | Acsl4 | mouse | Forward | CAATAGAGCAGAGTACCCTGAG |
|  |  | mouse | Reverse | TAGAACCACTGGTGTACATGAC |
|  | PGC-1α | human | Forward | CTGCGGGATGATGGAGACAG |
|  |  | human | Reverse | TTCGTTTGACCTGCGCAAAG |
|  | NRF2 | human | Forward | CAGCGACGGAAAGAGTATGA |
|  |  | human | Reverse | TGGGCAACCTGGGAGTAG |
|  | GAPDH | human | Forward | TCGGAGTCAACGGATTTGGT |
|  |  | human | Reverse | TTCCCGTTCTCAGCCTTGAC |
|  | Gapdh | mouse | Forward | CAGGAGGCATTGCTGATGAT |
|  |  | mouse | Reverse | GAAGGCTGGGGCTCATTT |
| ChIP-qPCR | GPX4 | human | Forward | TAGGCAACATAGCGAGACCC |
|  |  | human | Reverse | CTCAAGCGAACCTCCCGTCT |
